# Nanoparticle-Mediated Delivery of Irbesartan Induces Cardioprotection from Myocardial Ischemia-Reperfusion Injury by Antagonizing Monocyte-Mediated Inflammation

**DOI:** 10.1038/srep29601

**Published:** 2016-07-11

**Authors:** Yasuhiro Nakano, Tetsuya Matoba, Masaki Tokutome, Daiki Funamoto, Shunsuke Katsuki, Gentaro Ikeda, Kazuhiro Nagaoka, Ayako Ishikita, Kaku Nakano, Jun-ichiro Koga, Kenji Sunagawa, Kensuke Egashira

**Affiliations:** 1 Department of Cardiovascular Medicine, Kyushu University Graduate School of Medical Sciences, Fukuoka, Japan; 2 Department of Cardiovascular Research, Development, and Translational Medicine, Kyushu University Graduate School of Medical Sciences, Fukuoka, Japan

## Abstract

Myocardial ischemia-reperfusion (IR) injury limits the therapeutic effect of early reperfusion therapy for acute myocardial infarction (AMI), in which the recruitment of inflammatory monocytes plays a causative role. Here we develop bioabsorbable poly-lactic/glycolic acid (PLGA) nanoparticles incorporating irbesartan, an angiotensin II type 1 receptor blocker with a peroxisome proliferator-activated receptor (PPAR)γ agonistic effect (irbesartan-NP). In a mouse model of IR injury, intravenous PLGA nanoparticles distribute to the IR myocardium and monocytes in the blood and in the IR heart. Single intravenous treatment at the time of reperfusion with irbesartan-NP (3.0 mg kg^−1^ irbesartan), but not with control nanoparticles or irbesartan solution (3.0 mg kg^−1^), inhibits the recruitment of inflammatory monocytes to the IR heart, and reduces the infarct size via PPARγ-dependent anti-inflammatory mechanisms, and ameliorates left ventricular remodeling 21 days after IR. Irbesartan-NP is a novel approach to treat myocardial IR injury in patients with AMI.

Acute myocardial infarction (MI) is a major cause of death and heart failure worldwide^1^. In patients with ST-segment elevation acute MI, early reperfusion therapy is a standard strategy to limit MI size and has improved clinical outcomes over the past 3 decades. However, recent cohort studies suggest that the mortality of MI patients has not improved despite significant reductions in door-to-balloon time in the last decade^2^. It is well recognized that the reperfusion of coronary arteries paradoxically induces cardiomyocyte death, known as myocardial ischemia-reperfusion (IR) injury, for which several new therapeutic strategies are under investigation^3^. Myocardial IR induces the generation of reactive oxygen species, calcium overload, and rapid pH correction, all of which cause mitochondrial injury through the opening of the mitochondrial permeability transition pore (MPTP) and the activation of mitochondrial outer membrane permeabilization, leading to the necrosis and apoptosis of cardiomyocytes in the early phase (in several minutes) of IR injury. In the late phase of injury (over several hours), myocardial inflammation contributes to cardiomyocyte apoptosis and the healing of infarcted myocardium^3^,^4^,^5^. Recent reports suggest that the recruitment of inflammatory monocytes (Ly6C^high^CCR2^+^ in mice, CD14^high^CD16^−^ in humans) contributes to myocardial inflammation after MI^6^,^7^,^8^, and endogenous angiotensin II and monocyte chemotactic protein-1 (MCP-1), a ligand for CCR2, play important roles in this process, suggesting their roles as potential therapeutic targets for IR injury^9^,^10^,^11^.

In the present study, we employed irbesartan, an angiotensin II type 1 receptor (AT1R) blocker (ARB) that possesses a partial agonistic effect on a nuclear receptor peroxisome proliferator-activated receptor (PPAR)γ^12^, as a therapeutic agent for IR injury. Previous studies have demonstrated that the ARB candesartan reduces myocardial inflammation by reducing the expression of pro-inflammatory cytokines such as IL-6, TNF-α and MCP-1 in cardiomyocytes after MI independent of any antihypertensive effect^13^,^14^. Importantly, PPARγ activation primes peripheral monocytes into macrophages with an anti-inflammatory phenotype^15^ and reduces inflammatory chemokine expression and macrophage infiltration into injured organs^16^,^17^, which suggests the potential anti-inflammatory effects of PPARγ activation in myocardial IR injury.

Although experimental studies have reported significant reduction of MI with therapeutic strategies designed to inhibit the inflammatory process, clinical studies targeting inflammation have failed to demonstrate any impact on clinical outcome in reperfusion-STEMI patients^18^,^19^. These discrepancies in the efficacy of anti-inflammatory therapies are attributed to an insufficient local drug concentration during a limited interventional time window, while administered at the time of reperfusion. Therefore, from a clinical perspective, it is essential to develop a drug delivery system (DDS) that facilitates delivery of clinically approved drugs to the sites of IR injury during reperfusion, a clinically feasible time point. Recently, we have developed a nanoparticle-mediated DDS using bioabsorbable poly-(lactic-co-glycolic acid) (PLGA) nanoparticles^20^,^21^,^22^,^23^,^24^. Nano-sized materials accumulate in injured tissues, including IR myocardium, where vascular permeability is enhanced^23^,^25^,^26^,^27^ and were rapidly taken up by circulating monocytes and reticuloendothelial phagocytic organs after intravenous administration^11^,^22^. Thus, PLGA nanoparticles are a feasible DDS for myocardial IR injury targeting ischemic myocardium and inflammatory monocytes.

In the present study, we engineered PLGA nanoparticles that incorporate irbesartan (irbesartan-NP). We show that PLGA nanoparticles deliver the drug to the mononuclear phagocytic system and IR myocardium after intravenous injection at the time of reperfusion. We also demonstrate that irbesartan-NP limits IR injury by antagonizing the recruitment of Ly6C^high^CCR2^+^ inflammatory monocytes into the ischemic myocardium.

## Results

### Ischemia-reperfusion recruits neutrophils and monocytes into the heart

**Figure 1:**
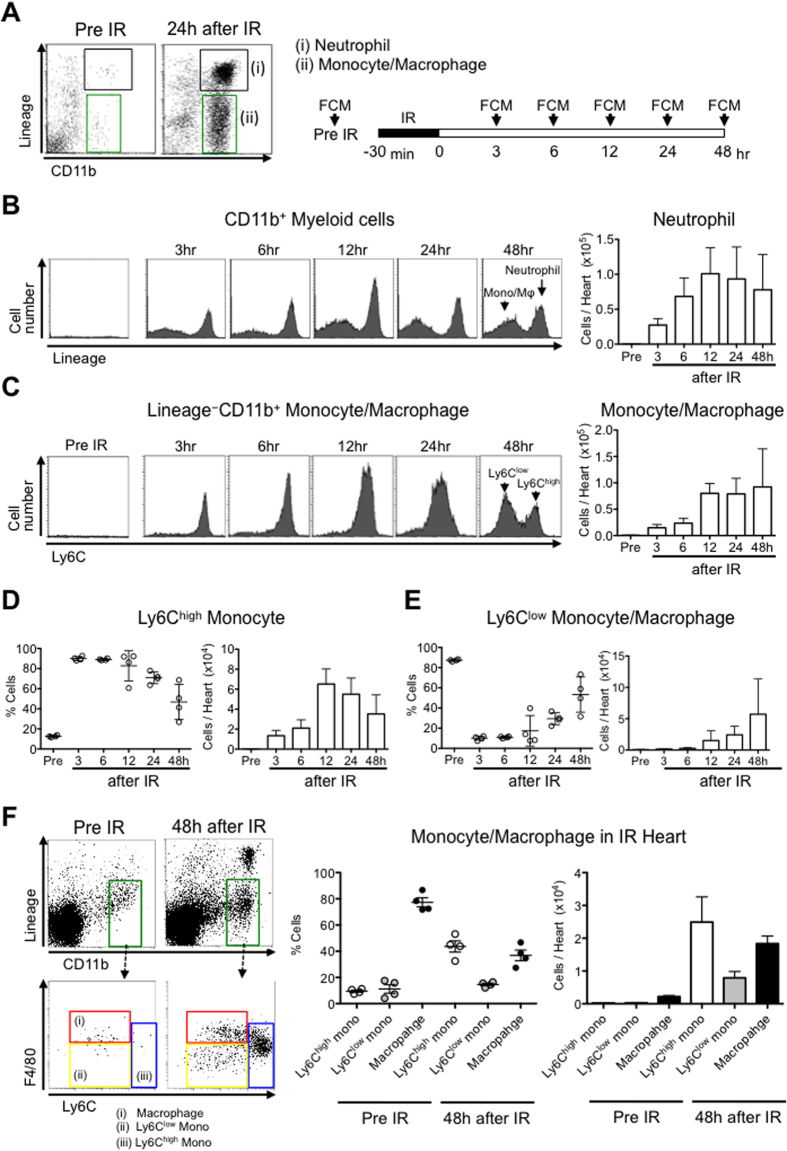
Ischemia-reperfusion mobilizes neutrophils and monocytes/macrophages to the heart. (**A**) Flow cytometric analysis of blood neutrophils and monocytes/macrophages. Monocytes/macrophages were identified as CD11b^high^Lineage^low^Ly6C^high/low^, and neutrophils were identified as CD11b^high^Lineage^high^. Lineage refers to the combination of CD90, B220, CD49b, NK1.1 and Ly6G monoclonal antibodies. (**B**) Representative histograms from individual mice within the CD11b^high^ Myeloid cells gate depict neutrophils and monocytes/macrophages in healthy hearts and within the heart at specified times after IR. Column graphs illustrate the total number of neutrophils per heart (N = 4 mice per group). (**C**) Representative histograms from individual mice within the monocytes/macrophages cell gate depict Ly6C^high^ and Ly6C^low^ monocytes in healthy hearts and within the heart at specified times after IR. Column graphs illustrate the total number of monocytes/macrophages per heart (N = 4 mice per group). (**D**) The percentage of Ly6C^high^ monocytes/macrophages and the total number of Ly6C^high^ monocytes/macrophages per heart. (**E**) The percentage of Ly6C^low^ monocytes/macrophages and the total number of Ly6C^low^ monocytes/macrophages per heart. (**F**) Flow cytometry of leukocytes in the healthy hearts and hearts 48 h after IR injury. Red gate shows the population of macrophages (CD11b^high^Lineage^low^F4/80^high^Ly6C^low^ cells). The percentage and the number of macrophages, Ly6C^high^ monocytes and Ly6C^low^ monocytes per CD11b^high^Lineage^low^ cells in IR heart. Data are expressed as the mean ± SEM (N = 4 mice). All results are average of three replicates. IR: ischemia reperfusion, FCM: flow cytometry, Mφ: macrophage, Mono: monocyte.

We induced IR injury in C57BL/6 mice and analyzed single-cell suspensions of digested IR heart at different time points (Fig. 1A). CD11b^+^Lineage^+^ neutrophils and CD11b^+^Lineage^−^ monocytes/macrophages were present in IR hearts at 3, 6, 12, 24 and 48 hours after reperfusion (Fig. 1B,C). Ly6C^high^ inflammatory monocytes dominated the monocyte population from 3 to 24 hours (Fig. 1D), whereas Ly6C^low^ monocytes populated from 24 to 48 hours after IR (Fig. 1E). We found that the majority of CD11b^+^Lineage^−^ cells are F4/80^+^Ly6C^low^ macrophages in the heart without ischemia (Fig. 1F). After 48 hours of reperfusion, the IR hearts were infiltrated with 44 ± 8% F4/80^−^Ly6C^high^ inflammatory monocytes, 15 ± 2% F4/80^−^Ly6C^low^ monocytes, and 37 ± 8% F4/80^+^Ly6C^low^ macrophages (Fig. 1F). The biphasic recruitment of the Ly6C^high^/Ly6C^low^ monocytes after myocardial IR was more rapid than that after MI^6^, and we could confirm the presence of Ly6C^high^ inflammatory monocytes in the heart tissues during the time of the late phase of IR injury in this murine model.

### Depletion of monocytes, but not neutrophils, reduced myocardial ischemia-reperfusion injury

**Figure 2:**
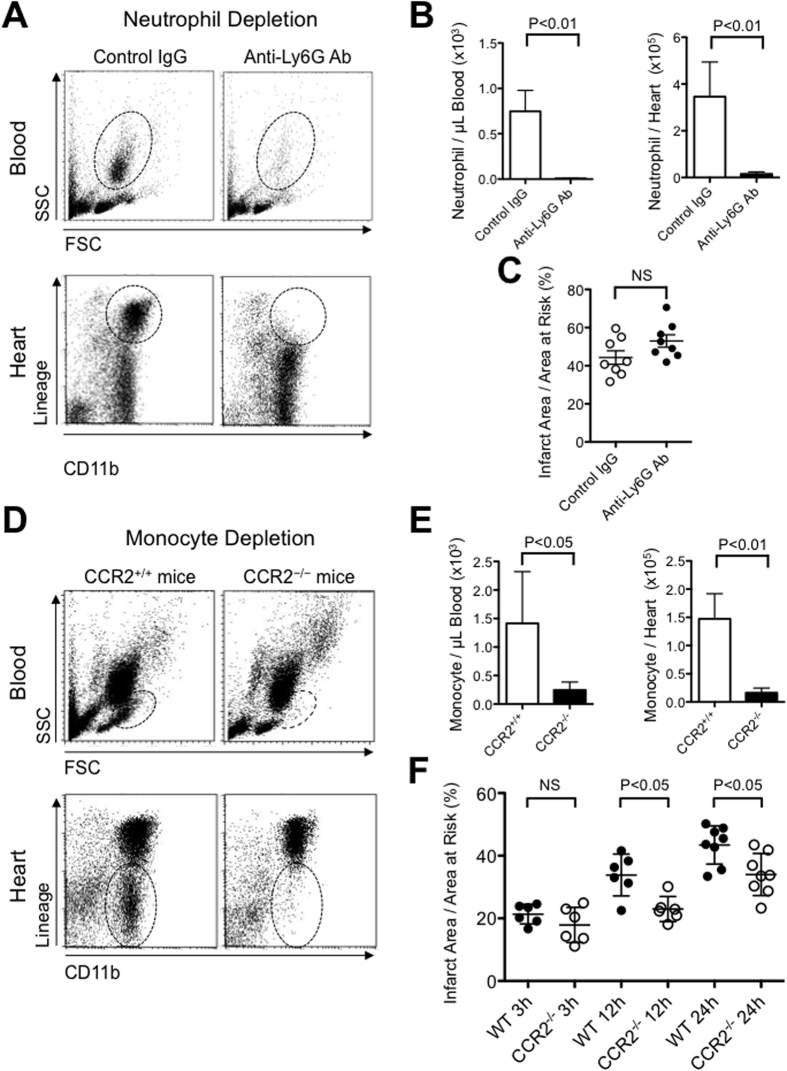
The role of neutrophil- or monocyte-mediated inflammation in myocardial IR injury. (**A,B**) The flow cytometric analysis demonstrates that pretreatment with an anti-Ly6G 1A8 monoclonal antibody decreased the number of neutrophils in the peripheral blood and the heart after 12 hours of reperfusion (N = 4 per group). (**C**) Pretreatment with anti-Ly6G antibody did not reduce infarct size 24 hours after reperfusion (N = 8 mice per group). (**D,E**) The number of monocytes in the blood and the heart after 12 hours of reperfusion was significantly reduced in CCR2^−/−^ mice compared with CCR2^+/+^ mice (N = 5 mice per group). (**F**) The time series of infarct size assessment after reperfusion of various durations (3 h, 12 h, 24 h after IR) on WT mice and CCR2^−/−^ mice (N = 6–8 mice per group). Data are expressed as the mean ± SD. Data are compared using the unpaired *t*-test. All results are average of three replicates. NS: not significant. SSC: side scatter, FSC: forward scatter.

To determine the role of neutrophils- or Ly6C^high^ monocytes-mediated inflammation in myocardial IR injury, we examined whether the depletion of neutrophils or monocytes reduces myocardial IR injury in a murine model. Intraperitoneal treatment with an anti-Ly6G 1A8 monoclonal antibody selectively depleted neutrophils from the peripheral blood without affecting other leukocytes in mice (Supplementary Fig. 1A). Pretreatment with anti-Ly6G decreased the numbers of neutrophils in the peripheral blood and the recruitment of neutrophils in the IR heart tissue 12 hours after reperfusion (Fig. 2A,B). Importantly, the inhibition of neutrophil recruitment did not reduce infarct size 24 hours after reperfusion in this murine model (Fig. 2C), suggesting that neutrophil may not be primary therapeutic target of IR injury in this model.

Because Ly6C^high^ inflammatory monocytes express high levels of CCR2 and because MCP-1/CCR2 signaling is a principal mediator of monocyte recruitment after MI^6^,^11^, we examined the role of Ly6C^high^ monocytes in IR injury by the use of CCR2^−/−^ mice. We confirmed that peripheral blood monocytes were significantly decreased in CCR2^−/−^ mice without IR (Supplementary Fig. 1B). Twelve hours after IR, the number of monocytes was significantly decreased in the blood and in the IR heart in CCR2^−/−^ mice (Fig. 2D,E). We examined a time series of infarct size after reperfusion of various durations (3h, 12h, and 24h) in WT and CCR2^−/−^ mice. There was no difference in infarct size between WT and CCR2^−/−^ mice after 3 hours of IR, when early phase of IR injury (e.g. MPTP opening) has taken place. Importantly, the infarct size enlarges over 24 hours, and the infarct size was smaller in CCR2^−/−^ mice at 12 hours and 24 hours after IR (Fig. 2F). These observations identify Ly6C^high^CCR2^+^ inflammatory monocyte, but not neutrophil, as a protagonist of cardiomyocyte death in the late phase of IR injury, and a potential therapeutic target for myocardial IR injury in this model.

### PLGA nanoparticle as a drug delivery system for IR injury

**Figure 3:**
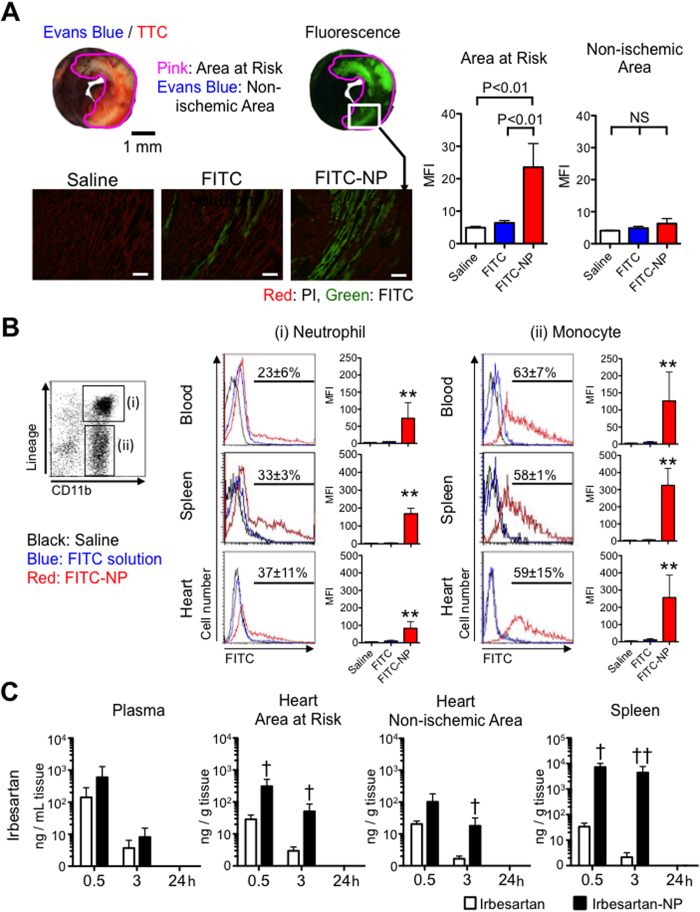
PLGA nanoparticles as a drug delivery system for IR injury. (**A**) Representative light (upper left) and fluorescence (upper right) stereomicrographs of whole hearts 3 hours after intravenous injection of FITC-NP. Fluorescence photomicrographs (lower panels) of cross-sections from hearts treated with saline, FITC solution and FITC-NP. Green indicates FITC, and red indicates nuclei (PI). Scale bar: 20 μm. Column graphs illustrate quantification of the FITC mean fluorescence intensity (MFI) in the area at risk and non-ischemic area. Data shown as mean ± SD (N = 4 mice per group). Data are compared using one-way ANOVA followed by Bonferroni’s multiple comparison tests. (**B**) Histograms illustrate the uptake of FITC-NP (red) in the respective cells (columns) and organs (rows). Column graphs illustrate quantification of the FITC MFI in the respective cells. Data shown as mean ± SD (N = 5 mice per group). The percentages of FITC-positive cells is reported as mean ± SEM (N = 3 mice per group). (**C**) Plasma, myocardial tissue (area at risk, non-ischemic area) and spleen concentrations of irbesartan after 30 minutes, 3 hours and 24 hours of intravenous injection of irbesartan alone or irbesartan-NP. Data shown as mean ± SD (N = 4–5 mice per group). *P < 0.05 vs. saline, **P < 0.01 vs. saline using one-way ANOVA followed by Bonferroni’s multiple comparison tests. ^†^P < 0.05 vs. irbesartan solution, ^††^P < 0.01 vs. irbesartan solution using two-way ANOVA followed by Bonferroni’s multiple comparison tests. All results are average of three replicates. NS: not significant.

To investigate the biodistribution of PLGA-NP, we injected indocyanine green-incorporated nanoparticles (ICG-NP) in mice and traced with *ex vivo* fluorescence reflectance imaging (FRI). FRI revealed that ICG did not distribute in the normal heart; importantly, the heart after IR exhibited high ICG presence (Supplementary Fig. 2A,B). ICG-NP also distributed in the liver, spleen and kidneys (Supplementary Fig. 2C). Histological analysis of the IR heart after the treatment with FITC-NP revealed that FITC-NP distributed in cardiomyocytes in the infarct area and in the area at risk (Fig. 3A). The magnified fluorescence microscopic images showed FITC signals in cardiomyocytes with clear cross striation (Supplementary Fig. 3). We next examined FITC signals in the leukocytes 6 hours after treatment with FITC solution or FITC-NP at the time of reperfusion. Flow cytometric analysis revealed the uptake of FITC-NP in the neutrophils and monocytes in the blood, spleen and heart (Fig. 3B). The incidence of FITC-positive cells was larger in monocytes (63 ± 7% in the blood, 59 ± 15% in the heart), than in neutrophils (23 ± 6% in the blood, 37 ± 11% in the heart). We also determined the plasma and tissue concentrations of irbesartan in the IR mouse model intravenously treated with irbesartan solution (3.0 mg kg^−1^) or irbesartan-NP (containing 3.0 mg kg^−1^ irbesartan) at the time of reperfusion. In the heart, irbesartan concentrations in the irbesartan-NP group were approximately 17-fold higher in ischemic myocardium compared with the irbesartan solution group 3 hours after IR (Fig. 3C). Irbesartan-NP attained approximately 3-hold higher irbesartan concentrations in ischemic versus non-ischemic myocardium, whereas irbesartan solution equivalently distributed in ischemic and non-ischemic myocardium (Supplementary Fig. 4, Fig. 3C). In the spleen, irbesartan-NP attained higher irbesartan concentrations compared with irbesartan solution (Fig. 3C). An *in vitro* analysis of irbesartan release kinetics showed an early burst of irbesartan release, in which approximately 90% of the total amount was released within 6 hours (Supplementary Fig. 5). These data indicate that PLGA nanoparticles are a feasible DDS for myocardial IR injury targeting ischemic myocardium and inflammatory monocytes in this model.

### Irbesartan-NP reduced myocardial IR injury by antagonizing monocyte-mediated inflammation

**Figure 4:**
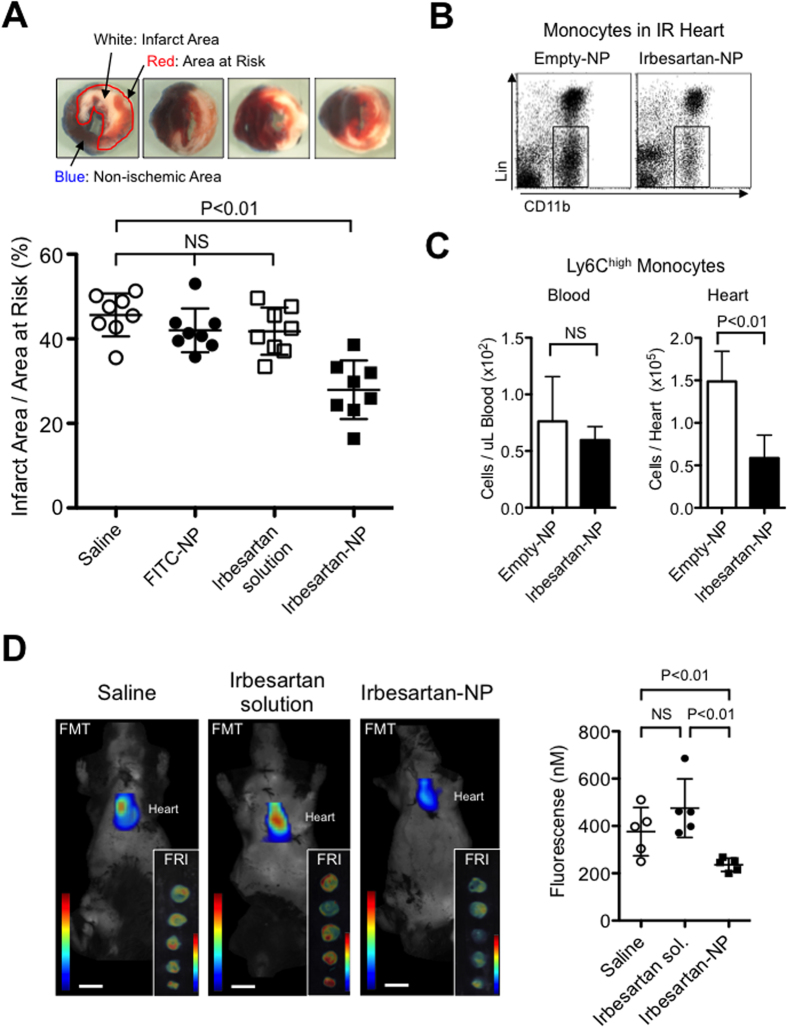
The anti-inflammatory effect of irbesartan-NP on IR injury. (**A**) The effects of saline, FITC-NP, irbesartan alone and irbesartan-NP on infarct size after IR. Data are expressed as the mean ± SD (N = 8 mice per group). Data are compared using one-way ANOVA followed by Bonferroni’s multiple comparison tests. (**B**) Flow cytometric analysis of the leukocytes in IR hearts treated with empty-NP and irbesartan-NP. (**C**) The number of Ly6C^high^ monocytes in the peripheral blood and the IR hearts after 12 hours of reperfusion. Data are expressed as the mean ± SD (N = 5 mice per group). Data are compared using the unpaired *t*-test. (**D**) Coronal FMT image acquired 24 hours after injection of Prosense-680 and demonstrates strong fluorescence in the cardiac region of IR mice. FRI imaging of the sections of heart 48 hours after IR. Scale bar: 10 mm. (**E**) Quantification of Prosense-680 activation 48 hours after IR. Data are reported as the mean ± SD (N = 5 mice per group). Data are compared using the ANOVA followed by Fisher’s *t*-test. All results are average of three replicates. FMT: fluorescence molecular tomography, FRI: fluorescence reflectance imaging, NS: not significant.

PLGA-NP-mediated delivery of irbesartan to monocytes and IR myocardium may enhance anti-inflammatory and cardioprotective effects of irbesartan for IR injury. Indeed, intravenous treatment with irbesartan-NP at the time of reperfusion (containing 3.0 mg kg^−1^ irbesartan) significantly reduced the infarct size 24 hours after reperfusion, but saline, FITC-NP or irbesartan solution (3.0 mg kg^−1^) was ineffective (Fig. 4A). We next examined the role of Ly6C^high^CCR2^+^ inflammatory monocytes in IR myocardium. Flow cytometric analysis 12 hours after reperfusion revealed that there were no differences in the number of total monocytes and Ly6C^high^CCR2^+^ inflammatory monocytes in the peripheral blood between two groups (Fig. 4B,C, Supplementary Fig. 6A). Importantly, irbesartan-NP significantly reduced the number of total monocytes and Ly6C^high^CCR2^+^ inflammatory monocytes in the heart (Fig. 4B,C, Supplementary Fig. 6B). These data suggested that irbesartan-NP inhibited the recruitment of monocytes to heart tissue from the peripheral blood.

We also performed *in vivo* fluorescence molecular tomography (FMT) imaging and *ex vivo* FRI to visualize and measure the protease activity in the heart after IR. We found that the inflammatory protease activity was markedly reduced in mice treated with irbesartan-NP (containing 3.0 mg kg^−1^ irbesartan) as compared with saline, whereas the same dose of irbesartan solution (3.0 mg kg^−1^) was ineffective, supporting the enhancement of drug efficacy by nanoparticle-mediated drug delivery (Fig. 4D). The infarct-limiting effect of irbesartan-NP was independent of antihypertensive effect, because the treatment with this dose of irbesartan-NP, saline or irbesartan solution did not affect systolic blood pressure after 6 and 24 hours of reperfusion (Supplementary Table 1). Echocardiography showed that irbesartan-NP, but not saline or irbesartan, ameliorated the enlargement of left ventricular dimension, and the decrease in left ventricular ejection fraction (LVEF) and LV fraction shortening (LVFS) 3-week after IR (Supplementary Table 2).

### Irbesartan-NP reduced myocardial IR injury via a PPARγ-dependent mechanism

**Figure 5:**
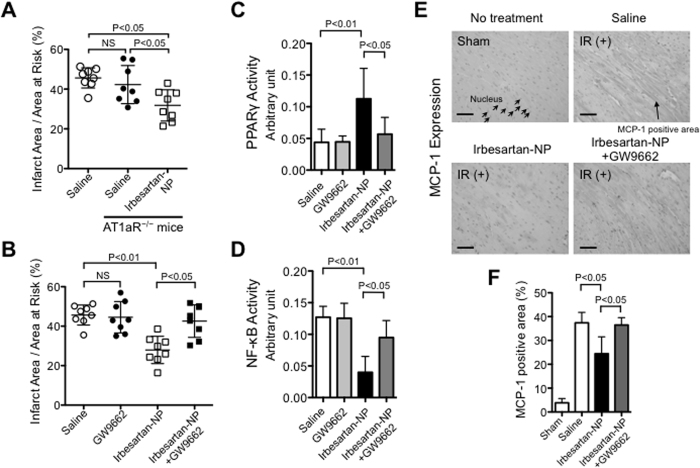
The therapeutic effect of irbesartan-NP was mediated largely through the PPARγ pathway. (**A**) The infarct size after IR between WT mice and AT1aR deficient mice and the effect of irbesartan-NP on IR injury in AT1aR deficient mice. Data are expressed as the mean ± SD (N = 8 mice per group). Data are compared using one-way ANOVA followed by Bonferroni’s multiple comparison tests. (**B**) The effects of the PPARγ antagonist GW9662 on the therapeutic effects of irbesartan-NP on MI size. Data are expressed as the mean ± SD (N = 8 mice per group). Data are compared using one-way ANOVA followed by Bonferroni’s multiple comparison tests. (**C**) PPARγ activation in ischemic myocardium 6 hours after reperfusion. Data are compared using one-way ANOVA followed by Bonferroni’s multiple comparison tests. (**D**) NF-κB activation in ischemic myocardium 6 hours after reperfusion. Data are compared using one-way ANOVA followed by Bonferroni’s multiple comparison tests. (**E**) Representative photomicrographs of cross-sections from IR myocardium stained with MCP-1. (**F**) Quantitative assessment of MCP-1 expression in ischemic myocardium 12 hours after reperfusion. Data are expressed as the mean ± SD (N = 6 mice per group). Data are compared using one-way ANOVA followed by Bonferroni’s multiple comparison tests. All results are average of three replicates. Scale bar: 20 μm. IR: ischemia reperfusion, MI: myocardial infarction, NS: not significant.

To explore the role of AT1R in cardioprotection, we examined the myocardial infarct size in WT and AT1aR-deficient mice and observed that the infarct size was equivalent between the WT and AT1aR-deficient mice 24 hours after IR (Fig. 5A). Interestingly, irbesartan-NP was nevertheless effective in reducing the infarct size even in AT1aR-deficient mice (Fig. 5A), suggesting an AT1aR-independent effect of irbesartan-NP. By contrast, the infarct-limiting effect of irbesartan-NP was abrogated by the pretreatment with a PPARγ antagonist GW9662 (Fig. 5B), highlighting a role of PPARγ activation. Irbesartan-NP activated PPARγ DNA binding in the nuclear extract from the ischemic myocardium, and systemic pretreatment with GW9662 prevented irbesartan-NP-induced PPARγ activation (Fig. 5C). PPARγ is known to repress NF-κB, which regulates inflammatory genes including MCP-1 in monocytes/macrophages^20^,^28^,^29^. Treatment with irbesartan-NP decreased NF-κB-DNA binding activity in the ischemic myocardium, which was reversed by the pretreatment with GW9662 (Fig. 5D). Immunohistochemical analysis revealed that irbesartan-NP reduced IR-induced MCP-1 expression in the area at risk, which was again reversed by GW9662 (Fig. 5E,F). These results suggested PPARγ-mediated anti-inflammatory mechanisms exerted by irbesartan-NP during the reduction of myocardial IR injury.

### The role of Ly6C^high^CCR2^+^ monocytes on the infarct-limiting effect of irbesartan-NP

**Figure 6:**
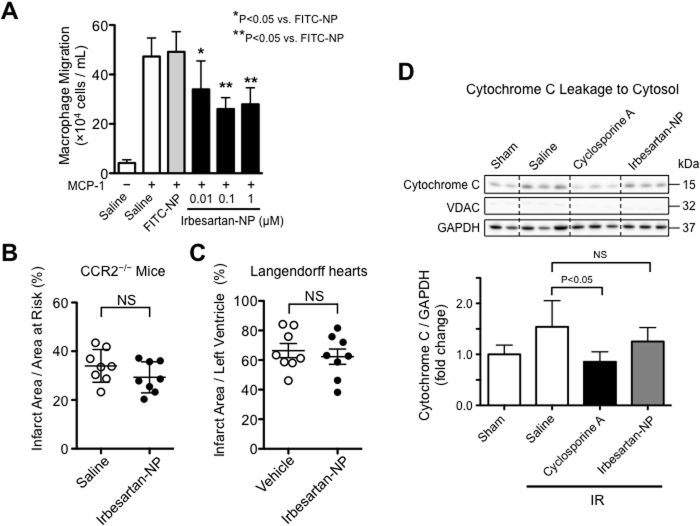
The role of inflammatory monocytes on the effects of irbesartan-NP. (**A**) The effects of irbesartan-NP on MCP-1 induced monocyte chemotaxis in THP-1 cells. Data are reported as the mean ± SD (N = 6 per group). Data are compared using one-way ANOVA followed by Bonferroni’s multiple comparison tests. (**B**) The effects of irbesartan-NP on IR injury in CCR2^−/−^ mice. Data are expressed as the mean ± SD (N = 8 mice per group). Data are compared using the unpaired *t*-test. (**C**) The effects of irbesartan-NP on Langendorff perfusion mice hearts subjected to ischemia and reperfusion. Data are expressed as the mean ± SD (N = 8 per group). Data are compared using the unpaired *t*-test. (**D**) The effects of irbesartan-NP on cytosolic cytochrome c in IR myocardium 30 minutes after reperfusion. Data are expressed as the mean ± SD (N = 6 mice per group). Data are compared using one-way ANOVA followed by Bonferroni’s multiple comparison tests. All results are average of three replicates. IR: ischemia reperfusion, NS: not significant.

We tested the chemotactic activity of treated THP-1 monocytes in response to MCP-1 by chemotaxis assay. Irbesartan-NP inhibited MCP-1-induced chemotaxis in a dose-dependent manner (Fig. 6A). Next, we examined the infarct-limiting effect of irbesartan-NP in CCR2^−/−^ mice that exhibit smaller infarct size compared with wild type mice (Fig. 2D–F). Importantly, irbesartan-NP did not show further reduction of IR injury in CCR2^−/−^ mice (Fig. 6B), indicating that CCR2-dependent recruitment of inflammatory monocytes is the therapeutic target of irbesartan-NP. We also examined the infarct-limiting effect of irbesartan-NP in Langendorff perfusion mice hearts subjected to ischemia and reperfusion. There were no significant differences between vehicle group and irbesartan-NP group in Langendorff perfusion hearts (Fig. 6C), supporting the notion that irbesartan-NP reduced myocardial IR injury through the inhibition of monocyte-mediated inflammation. Finally, we examined the interaction of mitochondria injury with cardioprotection by irbesartan-NP. Treatment with irbesartan-NP did not reduce cytochrome c leakage from the mitochondria into the cytosol 60 minutes after IR, whereas pretreatment with cyclosporine A, an inhibitor of the MPTP, significantly reduced cytochrome c release into cytosolic fraction after IR (Fig. 6D). These data suggest that the therapeutic effects of irbesartan-NP are independent of the mitochondrial pathway of IR injury that might contribute to the early phase of myocardial IR injury.

## Discussion

The novel findings of the present study are as follows; 1) Ly6C^high^CCR2^+^ monocytes, but not neutrophils, play a detrimental role in myocardial IR injury in a mouse model; 2) PLGA-NP delivers a drug to the mononuclear phagocytic system and IR myocardium after intravenous injection at the time of reperfusion; and 3) PLGA nanoparticle-mediated delivery of irbesartan protects the hearts from IR injury via PPARγ-dependent anti-inflammatory mechanisms and ameliorated left ventricular remodeling.

Myocardial IR initiates several molecular mechanisms such as a production of reactive oxygen species, a calcium overload, and a pH correction, which lead to mitochondrial injury and myocardial hypercontracture^30^. Mitochondrial injury mediated by the opening of MPTP is a major mechanism of myocardial IR injury in the early phase of IR injury. MPTP opening causes the rupture of mitochondrial membrane, cytochrome c leakage to the cytosol, and cardiomyocyte deaths; therefore, mitochondrial injury has been an important therapeutic target of the treatment for myocardial IR injury. Indeed, a phase 3 Does Cyclosporine Improve Clinical Outcome in ST Elevation Myocardial Infarction Patients (CIRCUS) trial has been carried out recently in patients with anterior ST-elevation acute MI who had been referred for primary percutaneous coronary intervention, although the treatment with intravenous cyclosporine, an inhibitor of MPTP, did not result in better clinical outcomes^31^.

Leukocytes, especially neutrophils and monocytes, are recruited to the areas of injury immediately after ischemia^32^ and accumulate into the myocardium after IR in several animal models and myocardial infarction in humans^6^,^8^,^33^. The contribution of inflammation to myocardial IR injury, however, has been questioned because of the failures of anti-inflammatory therapies (Supplementary Table 3). In this study, we could confirm the recruitment of neutrophils and Ly6C^high^CCR2^+^ inflammatory monocytes 3–24 hours after IR, followed by Ly6C^low^ monocytes (24–48 hours after IR) in the mouse model used in this study (Fig. 1). Previous studies have demonstrated the contribution of neutrophils in myocardial IR injury by the use of a leukocyte filter, polymorphonuclear neutrophils (PMN) antiserum, antibodies against CD18, CD11, intercellular adhesion molecule (ICAM)-1, P-selectin, Saily Lewis^x^ Analogue, cyclooxygenase inhibitors, and other methods (Supplementary Table 3). However, these interventions are not specific to neutrophils, and the most target molecules are also expressed in monocytes. Three studies by Romson^34^, Jolly^35^, and Chatelain^33^ have reported neutrophil depletion without affecting monocytes using anti-PMN serum in canine myocardial IR models; however, the reduction of infarct size was inconsistent (Supplementary Table 3). Therefore, the role of neutrophils in IR injury remains controversial^36^. In this study, antibodies against Ly6G, a neutrophil-specific differentiation marker, depleted neutrophils without affecting other types of leukocytes in mice (Supplementary Fig. 1A), in accordance with previous reports^37^. Importantly, treatment with an anti-Ly6G antibody reduced neutrophil infiltration into the heart after IR but did not reduce the infarct size, suggesting a negligible role of neutrophils in IR injury in the mouse model used in this study (Fig. 2A–C).

Recent reports suggest that the recruitment of Ly6C^high^ inflammatory monocytes contributes to myocardial inflammation after MI^6^,^7^,^38^. In this study, we found that Ly6C^high^ monocytes dominate (approximately 80%) the monocytes recruited into the heart during first 24 hours of IR injury (Fig. 1D,E) and that CCR2 deficiency reduced the recruitment of Ly6C^high^ monocytes by approximately 90% and reduced the infarct size as well (Fig. 2E,F). Time series analysis of infarct size (Fig. 2F) showed delayed enlargement of infarct size and the differences in the infarct size between CCR2^−/−^ and WT mice at 12 and 24 hours after IR, which suggest the contribution of monocyte-mediate inflammation to myocardial IR injury in the late phase. These observations suggest that Ly6C^high^CCR2^+^ inflammatory monocytes, but not neutrophils, are the central therapeutic target for myocardial inflammation and IR injury in this model. Relative contributions of different mechanisms of myocardial injury including mitochondrial injury and inflammation need to be clarified in a future study.

FITC-loaded PLGA nanoparticles were incorporated in circulating neutrophils and monocytes and delivered preferentially into ischemic myocardium (Fig. 3A,B, Supplementary Fig. 3). The incidence of FITC-positive cells was larger in monocytes than in neutrophils 6 hours after intravenous administration (Fig. 3B). The differences may reflect a shorter half-life of neutrophil (approximately 6 hours)^39^ and monocytes (more than days)^40^. Monocytes exhibited a prominent uptake of FITC-NP, increasing the FITC fluorescence intensity in the heart, approximately 25-fold compared with FITC solution (Fig. 3B). Along with FITC-NP distribution, irbesartan concentrations determined in mice treated with irbesartan solution or irbesartan-NP (Fig. 3C) suggest that PLGA nanoparticles modulate the *in vivo* kinetics of irbesartan through a delivery to ischemic cardiomyocytes via enhanced vascular permeability^25^,^27^. Nanoparticles are also taken up by mononuclear phagocytic systems, including circulating neutrophils and monocytes, and splenic monocytes/macrophages that are potential sources of Ly6C^high^ monocytes recruited to IR myocardium^7^,^11^. We have previously demonstrated that monocytes/macrophages exhibited strong uptake of FITC-NP *in vivo* and *in vitro*^22^. Thus, PLGA nanoparticles are a feasible DDS to target the ischemic myocardium and inflammatory monocytes after IR, with payloads for cardioprotection.

To translate this nanotechnology-based DDS into clinical medicine, we developed PLGA nanoparticles loaded with irbesartan, an ARB with partial PPARγ agonistic activity, to regulate inflammation after myocardial IR. A previous study reported that small interfering RNAs (siRNAs) against CCR2 delivered in lipid nanoparticles can experimentally reduce myocardial infarct size in mouse model of myocardial ischemia reperfusion^11^, suggesting the feasibility of targeting CCR2^+^ inflammatory monocytes; however, chemicals including irbesartan possess superior stability to siRNA especially *in vivo*, raising the possibility of clinical development. From a translational aspect, the timing of administration is also important. Several animal studies employed the pretreatment of therapeutic agents before the induction of IR^5^, whereas the pretreatment is often difficult to perform in clinical trials and practice. In this study, we demonstrated that the intravenous injection of PLGA nanoparticles at the time of reperfusion is a feasible DDS for the treatment of myocardial IR injury. Nanoparticulation attained approximately 17 -fold higher irbesartan concentrations in the IR myocardium 30-min after reperfusion and enhanced cardioprotective effects of irbesartan, proving the concept. A 7-day pretreatment with oral irbesartan (50 mg kg^−1^ day^−1^)^41^ reduced the infarct size by a similar degree with a single injection of irbesartan-NP (Supplementary Fig. 7), supporting the efficacy of PLGA nanoparticles as a DDS.

PLGA nanoparticles are well-established drug carriers that optimize pharmacokinetics with biosafety approval for human use by the US Food and Drug Administration (FDA)^27^,^42^. PLGA nanoparticles have been tested as a DDS for tumors previously^27^,^43^. In those studies, uptake of PLGA nanoparticles by leukocytes in reticulo-endothelial organs reduced circulating time and delivery to tumors, which were overcome by surface modification with poly (ethylene glycol). In this study, PLGA nanoparticles without surface modification were delivered to circulating and splenic leukocytes as well as ischemic myocardium. As spleen is a source of monocyte recruitment to injured myocardium^38^, this passive delivery of PLGA nanoparticles was purposive as a DDS for myocardial IR injury.

We employed emulsion solvent diffusion method to produce irbesartan-NP that does not modify the chemical structures of incorporating irbesartan and PLGA. Although PLGA nanoparticles clearly increase irbesartan concentration in the ischemic myocardium and the spleen, and enhanced therapeutic efficacy of irbesartan, this composition of nanoparticle may compromise the specificity of delivery and the retardation of drug release, potential advantages of PLGA nanoparticle as a drug carrier^27^,^43^. From *in vitro* drug release study, approximately 90% of irbesartan was released to physiological solution within 6 hours (Supplementary Fig. 5), before decomposition of PLGA nanoparticles via hydrolysis, which takes approximately 14 days at 32°C and pH 7.4^44^. Although one of the original purposes of nanoscale drug delivery system was a slow release of its content, PLGA nanoparticles exhibits initial fast release of the content^27^, and the foregoing fast release of irbesartan was reasonable for the treatment of AMI, in which mitochondrial injury and the initiation of inflammatory response occurs within several minutes. We also took advantage of its delivery to phagocytes, which was originally considered as a disadvantage for the treatment of cancers^27^, but now found effective to regulate monocyte-mediated inflammation in myocardial ischemia-reperfusion injury in AMI. Another rationale of the use of this nanoparticle composition is excellent safety profiles of unmodified irbesartan, an approved antihypertensive drug, and PLGA in the development as a new drug. Indeed, PLGA nanoparticles containing pitavastatin (an HMG-CoA reductase inhibitor) produced with the same process has been investigated by the Japanese regulatory agency (Pharmaceuticals and Medical Devices Agency, PMDA), and we have started phase I/IIa investigator’s initiated clinical trial in the Kyushu University Hospital (clinical trial registry ID: UMIN000008011), investigating the efficacy of PLGA nanoparticle-mediated delivery of pitavastatin in patients with critical limb ischemia. Irbesartan is clinically approved and has been widely used for the treatment of hypertension and hypertension-related cardiovascular end-organ diseases. Therefore, irbesartan-NP is a new therapeutic agent with high efficacy and safety for myocardial IR injury, which is readily translatable into clinical trials.

In this study, Irbesartan-NP did not reduce cytochrome c leakage from mitochondria to the cytosol 60 minutes after IR, which was reduced by a pretreatment with cyclosporine A that binds to cyclophilin D, a critical component of MPTP (Fig. 6D). Additionally, irbesartan-NP failed to show direct protective effect in Langendorff perfusion hearts (Fig. 6C), excluding the possibility that irbesartan-NP may act on MPTP opening-associated cardiomyocyte death. Targeting inflammation that contributes in the late phase may widen therapeutic window, however, earliest administration of irbesartan-NP might be beneficial, because the infiltration of inflammatory cells starts immediately after ischemia^32^. Myocardial inflammation contributes to cardiomyocyte apoptosis and the healing of infarcted myocardium in the late phase of IR injury. In this study, cardioprotection by irbesartan-NP was associated with an inhibition of Ly6C^high^CCR2^+^ inflammatory monocytes recruitment into IR myocardium.

The infarct-limiting effect of irbesartan-NP was abrogated by the pretreatment with a PPARγ antagonist GW9662 (Fig. 5B), indicating that irbesartan-NP reduced myocardial IR injury through mainly PPARγ pathway. Irbesartan-NP reduced MCP-1 expression in the myocardium (Fig. 5E,F) and inhibited chemotactic activity toward MCP-1 in cultured monocytes (Fig. 6A), both of which are attributable to PPARγ-mediated transrepression of NF-κB^28^,^29^. Importantly, irbesartan-NP was no longer effective in CCR2^−/−^ mice and Langendorff perfusion hearts (Fig. 6B,C), suggesting that the inhibition of Ly6C^high^CCR2^+^ inflammatory monocytes recruitment is a primary mechanism of cardioprotection by irbesartan-NP. PPARγ is known to regulate macrophage polarity toward Ly6C^low^ anti-inflammatory monocytes/macrophages, which is potentially relevant to inflammation healing. A recent study by the authors’ group has tested the effects of PLGA nanoparticles containing a PPAR-γ agonist pioglitazone in an atherosclerotic mouse model and found that pioglitazone-nanoparticles induced M2 macrophage polarity shift in the atherosclerotic lesions^45^. Irbesartan-NP might also induce Ly6C^low^ monocytes/anti-inflammatory M2 macrophages, and facilitate inflammation resolution and healing in this model. However, flow cytometric analysis 12 hours after reperfusion revealed that there were no differences in the number of Ly6C^low^ monocytes/macrophages in the IR myocardium between two groups (Supplementary Fig. 6).

A previous study^38^ has shown the role of AT1 receptor in monocyte recruitment from spleen after experimental myocardial infarction in mice, for which pretreatment with angiotensin converting enzyme inhibitor enalapril or ARB losartan reduced monocyte recruitment. Rodents express AT1a and AT1b. AT1a is dominant over AT1b in most cardiovascular tissues^46^, therefore, AT1a-deficient mice are widely used to characterize cardiovascular effects of angiotensin receptor blockers. In this study on myocardial IR injury, we found that the infarct size was unaffected by AT1aR deficiency, and that the infarct-limiting effect of irbesartan-NP was independent of AT1aR blockade, as indicated from the experiments in AT1aR^−/−^ mice (Fig. 5). The difference may owe to the different degrees of myocardial injury and thereby proinflammatory mediators (damage-associated molecular patterns, chemokines, angiotensin II, etc.)^38^,^47^. Previous study has reported that infarct size reduction by candesartan through a cascade of AT2 receptor activation in pig myocardial IR model^48^. Although the therapeutic effect of irbesartan-NP was mediated by CCR2, and was independent of AT1aR in this study (Fig. 5), the contribution of AT2R in monocyte mobilization may explain the different migration patterns of Ly6C^high/low^ monocytes, and may be a potential therapeutic target of nanoparticle-mediated drug delivery. Further study is needed to clarify the role of AT2R. The inhibition of left ventricular remodeling by irbesartan-NP may be attributed to the reduction of infarct size itself, and AT1R blockade^49^ by irbesartan-NP, although the relative importance of these mechanisms is unclear.

There are limitations in the present study. First, mice are not a suitable system to assess microvascular dysfunction or the ‘no-reflow phenomenon’ during IR injury, which may alter reperfusion itself as well as infarct size in large animal models and humans^50^. Preclinical studies are needed in large animals to proceed to the investigation of irbesartan-NP in humans^51^. Second, we only adopted the protocol of intravenous administration of irbesartan at the indicated dose. This dose of irbesartan (3.0 mg kg^−1^) is the maximum dose that can be administered intravenously in this mouse model due to its solubility. Preclinical studies are needed in large animals to determine the dose for optimal safety and efficacy of irbesartan-NP. Third, FITC-NP was prepared without chemical conjugation of fluorescein and PLGA, which might compromise the specificity of the tracing of PLGA nanoparticles by the release of FITC from PLGA nanoparticles. FITC-NP qualitatively characterized the distribution of PLGA nanoparticles in leukocytes in the circulation, spleen and IR heart by histology and flow cytometry (Fig. 3A,B), and the preferential distribution of PLGA nanoparticles in IR myocardium compared to non-ischemic myocardium was confirmed by tissue concentration of irbesartan (Fig. 3C).

In conclusion, this study identified inflammatory monocytes as an essential therapeutic target in myocardial IR injury, and demonstrated the feasibility of PLGA nanoparticle-mediated DDS for the treatment of myocardial IR injury, and the proof-of-concept results of irbesartan-NP in a murine model of myocardial IR injury. This nanoparticle-based technology may advance current unsatisfactory reperfusion therapy for AMI.

## Methods

Additional details of the experimental procedure are provided in the online-only Data Supplement.

### Mouse myocardial IR injury model

Adult male C57BL/6J mice (9–13 weeks old) (CLEA Japan, Inc), AT1aR-defecient mice on C57BL/6J background (gift from Dr. Hiroyuki Yamada, Kyoto Prefectural University School of Medicine)^52^ and CCR2^−/−^ mice on C57BL/6J and 129/svjae hybrids background^53^ were used in this study. The myocardial IR model was based on previously described methods^54^,^55^. Additional details are provided in the online-only Data Supplement.

### Experimental protocol

The study protocol was reviewed and approved by the Committee on the Ethics of Animal Experiments, Kyushu University Graduate School of Medical Science and was conducted in accordance with the Animal Physiological Society guidelines. The 8 sets of animal experiments are described in the online-only Data Supplement.

### Preparation of PLGA nanoparticles

PLGA nanoparticles encapsulated with fluorescein isothiocyanate (FITC; Dojin Chemical, Tokyo, Japan) (FITC-NP), indocyanine green (ICG; Tokyo Chemical Industry Co. Ltd., Tokyo, Japan) (ICG-NP) and irbesartan (Shionogi & Co. Ltd., Osaka, Japan) (irbesartan-NP) were prepared using an emulsion solvent diffusion method, as previously reported^22^,^56^.

Additional details can be found in the online-only Data Supplement.

### Distribution of FITC-nanoparticles

The distribution of FITC-NP in the peripheral blood leukocytes was analyzed with a FACSCalibur cytometer (Becton Dickinson Biosciences). The heart was harvested and fixed in 10% phosphate-buffered formalin (pH 7.4), and the distribution of FITC-NP was analyzed in 5-μm OCT-embedded sections. Additional details can be found in the online-only Data Supplement.

### Histological and immunohistochemical analyses

Histological and immunohistochemical evaluations were performed to examine the PPARγ-dependent anti-inflammatory mechanisms. The ischemic hearts after reperfusion were stained MCP-1. Additional details are provided in the online-only Data Supplement.

### Western blot analysis

Homogenates of IR myocardium were analyzed with immunoblotting. At predetermined time points, ischemic myocardium was isolated and analyzed as previously reported^57^. Additional details can be found in the online-only Data Supplement

### PPARγ and NF-κB activity in myocardium

Nuclear extracts were prepared from the myocardium homogenates using a nuclear extract kit (NE-PER Nuclear and Cytoplasmic Extraction Reagents; Thermo Fisher Scientific Inc., Rockford, IL) according to the manufacturer’s instructions. The protein was measured using a BCA Protein Assay kit (Thermo Fisher Scientific Inc.). PPARγ and NF-κB activation were assayed using an ELISA-based PPARγ activation TransAM kit (Active Motif, Rixensart, Belgium) and an ELISA-based NF-κB activation TransAM kit (Active Motif, Rixensart, Belgium), which were used according to the manufacturer’s instructions. Additional details are provided in the online-only Data Supplement.

### Flow cytometry

Leukocytes from peripheral blood, the heart and spleen were obtained from mice and analyzed with FACS Calibur (BD Biosciences, San Jose, CA, USA). Additional details are described in the online-only Data Supplement.

### Measurements of irbesartan concentrations in the plasma and tissues

Irbesartan concentrations in the plasma and tissues were measured at predetermined time points by liquid chromatography coupled to tandem mass spectrometry (LC/MS/MS). Additional details can be found in the online-only Data Supplement.

### Chemotaxis assay

The chemotactic activity of THP-1 cells in response to MCP-1 was measured in a 96-well microchemotaxis Boyden chamber (ChemoTx; Neuroprobe), as described previously^58^. Additional details are provided in the online-only Data Supplement.

### Statistical analysis

Data are expressed as the mean ± SD or SEM. Differences were statistically analyzed by ANOVA followed by post-hoc Bonferroni’s multiple comparison tests. Differences between the two groups were analyzed with using an unpaired *t-test.* Any *P* value less than 0.05 was considered significant.

## Additional Information

**How to cite this article**: Nakano, Y. *et al*. Nanoparticle-Mediated Delivery of Irbesartan Induces Cardioprotection from Myocardial Ischemia-Reperfusion Injury by Antagonizing Monocyte-Mediated Inflammation. *Sci. Rep.* **6**, 29601; doi: 10.1038/srep29601 (2016).

## Supplementary Material

Supplementary Information

## References

[b1] Go A. S. . Heart disease and stroke statistics–2013 update: a report from the American Heart Association. Circulation 127, e6–e245 (2013). 23239837 10.1161/CIR.0b013e31828124ad PMC5408511

[b2] Menees D. S. . Door-to-balloon time and mortality among patients undergoing primary PCI. N. Engl. J. Med. 369, 901–909 (2013). 24004117 10.1056/NEJMoa1208200

[b3] Hausenloy D. J. & Yellon D. M. Myocardial ischemia-reperfusion injury: a neglected therapeutic target. J. Clin. Invest. 123, 92–100 (2013). 23281415 10.1172/JCI62874 PMC3533275

[b4] Marchant D. J. . Inflammation in myocardial diseases. Circ. Res. 110, 126–144 (2012). 22223210 10.1161/CIRCRESAHA.111.243170

[b5] Vander Heide R. S. & Steenbergen C. Cardioprotection and myocardial reperfusion: pitfalls to clinical application. Circ. Res. 113, 464–477 (2013). 23908333 10.1161/CIRCRESAHA.113.300765 PMC3824252

[b6] Nahrendorf M. . The healing myocardium sequentially mobilizes two monocyte subsets with divergent and complementary functions. J. Exp. Med. 204, 3037 (2007). 18025128 10.1084/jem.20070885 PMC2118517

[b7] Swirski F. K. . Identification of splenic reservoir monocytes and their deployment to inflammatory sites. Science 325, 612–616 (2009). 19644120 10.1126/science.1175202 PMC2803111

[b8] Tsujioka H. . Impact of heterogeneity of human peripheral blood monocyte subsets on myocardial salvage in patients with primary acute myocardial infarction. J. Am. Coll. Cardiol. 54, 130–138 (2009). 19573729 10.1016/j.jacc.2009.04.021

[b9] Hayasaki T. . CC chemokine receptor-2 deficiency attenuates oxidative stress and infarct size caused by myocardial ischemia-reperfusion in mice. Circ. J. 70, 342 (2006). 16501303 10.1253/circj.70.342

[b10] Liehn E. A. . A New Monocyte Chemotactic Protein-1/Chemokine CC Motif Ligand-2 Competitor Limiting Neointima Formation and Myocardial Ischemia/Reperfusion Injury in Mice. J. Am. Coll. Cardiol. 56, 1847–1857 (2010). 21087715 10.1016/j.jacc.2010.04.066

[b11] Leuschner F. . Therapeutic siRNA silencing in inflammatory monocytes in mice. Nat. Biotechnol. 29, 1005–1010 (2011). 21983520 10.1038/nbt.1989 PMC3212614

[b12] Schupp M., Janke J., Clasen R., Unger T. & Kintscher U. Angiotensin type 1 receptor blockers induce peroxisome proliferator-activated receptor-gamma activity. Circulation 109, 2054–2057 (2004). 15117841 10.1161/01.CIR.0000127955.36250.65

[b13] Kohno T. . Angiotensin-receptor blockade reduces border zone myocardial monocyte chemoattractant protein-1 expression and macrophage infiltration in post-infarction ventricular remodeling. Circ. J. 72, 1685–1692 (2008). 18753699 10.1253/circj.cj-08-0115

[b14] Jugdutt B. I. . Aging-related early changes in markers of ventricular and matrix remodeling after reperfused ST-segment elevation myocardial infarction in the canine model: effect of early therapy with an angiotensin II type 1 receptor blocker. Circulation 122, 341–351 (2010). 20625108 10.1161/CIRCULATIONAHA.110.948190

[b15] Bouhlel M. A. . PPARγ Activation Primes Human Monocytes into Alternative M2 Macrophages with Anti-inflammatory Properties. Cell Metabolism 6, 137–143 (2007). 17681149 10.1016/j.cmet.2007.06.010

[b16] Odegaard J. I. . Macrophage-specific PPARγ controls alternative activation and improves insulin resistance. Nature 447, 1116–1120 (2007). 17515919 10.1038/nature05894 PMC2587297

[b17] Ahmadian M. . PPARγ signaling and metabolism: the good, the bad and the future. Nat. Med. 19, 557–566 (2013). 23652116 10.1038/nm.3159 PMC3870016

[b18] Armstrong P. W. . Pexelizumab for acute ST-elevation myocardial infarction in patients undergoing primary percutaneous coronary intervention: a randomized controlled trial. JAMA 297, 43 (2007). 17200474 10.1001/jama.297.1.43

[b19] Atar D. . Effect of intravenous FX06 as an adjunct to primary percutaneous coronary intervention for acute ST-segment elevation myocardial infarction results of the F.I.R.E. (Efficacy of FX06 in the Prevention of Myocardial Reperfusion Injury) trial. J. Am. Coll. Cardiol. 53, 720–729 (2009). 19232907 10.1016/j.jacc.2008.12.017

[b20] Kimura S. . Nanoparticle-mediated delivery of nuclear factor kappaB decoy into lungs ameliorates monocrotaline-induced pulmonary arterial hypertension. Hypertension 53, 877–883 (2009). 19307469 10.1161/HYPERTENSIONAHA.108.121418

[b21] Nagahama R. . Nanoparticle-mediated delivery of pioglitazone enhances therapeutic neovascularization in a murine model of hindlimb ischemia. Arterioscler. Thromb. Vasc. Biol. 32, 2427–2434 (2012). 22879581 10.1161/ATVBAHA.112.253823

[b22] Katsuki S. . Nanoparticle-Mediated Delivery of Pitavastatin Inhibits Atherosclerotic Plaque Destabilization/Rupture in Mice by Regulating the Recruitment of Inflammatory Monocytes. Circulation 129, 896–906 (2014). 24305567 10.1161/CIRCULATIONAHA.113.002870

[b23] Nagaoka K. . A New Therapeutic Modality for Acute Myocardial Infarction: Nanoparticle-Mediated Delivery of Pitavastatin Induces Cardioprotection from Ischemia-Reperfusion Injury via Activation of PI3K/Akt Pathway and Anti-Inflammation in a Rat Model. PLoS ONE 10, e0132451 (2015). 26167913 10.1371/journal.pone.0132451 PMC4500569

[b24] Ikeda G. . Nanoparticle-Mediated Targeting of Cyclosporine A Enhances Cardioprotection Against Ischemia-Reperfusion Injury Through Inhibition of Mitochondrial Permeability Transition Pore Opening. Sci. Rep. 6, 20467 (2016). 26861678 10.1038/srep20467 PMC4748220

[b25] Dauber I. M. . Functional coronary microvascular injury evident as increased permeability due to brief ischemia and reperfusion. Circ. Res. 66, 986–998 (1990). 2180590 10.1161/01.res.66.4.986

[b26] Takahama H. . Prolonged targeting of ischemic/reperfused myocardium by liposomal adenosine augments cardioprotection in rats. J. Am. Coll. Cardiol. 53, 709–717 (2009). 19232905 10.1016/j.jacc.2008.11.014

[b27] Acharya S. & Sahoo S. K. PLGA nanoparticles containing various anticancer agents and tumour delivery by EPR effect. Adv. Drug Deliv. Rev. 63, 170–183 (2011). 20965219 10.1016/j.addr.2010.10.008

[b28] Pascual G. . A SUMOylation-dependent pathway mediates transrepression of inflammatory response genes by PPAR-γ. Nature 437, 759–763 (2005). 16127449 10.1038/nature03988 PMC1464798

[b29] Duan S. Z., Usher M. G. & Mortensen R. M. Peroxisome proliferator-activated receptor-gamma-mediated effects in the vasculature. Circ. Res. 102, 283–294 (2008). 18276926 10.1161/CIRCRESAHA.107.164384

[b30] Frohlich G. M., Meier P., White S. K., Yellon D. M. & Hausenloy D. J. Myocardial reperfusion injury: looking beyond primary PCI. Eur. Heart J. 34, 1714–1722 (2013). 23536610 10.1093/eurheartj/eht090

[b31] Cung T. T. . Cyclosporine before PCI in Patients with Acute Myocardial Infarction. N. Engl. J. Med. 373, 1021–1031 (2015). 26321103 10.1056/NEJMoa1505489

[b32] Jung K. . Endoscopic time-lapse imaging of immune cells in infarcted mouse hearts. Circ. Res. 112, 891–899 (2013). 23392842 10.1161/CIRCRESAHA.111.300484 PMC3834270

[b33] Chatelain P. . Neutrophil accumulation in experimental myocardial infarcts: relation with extent of injury and effect of reperfusion. Circulation 75, 1083–1090 (1987). 3568308 10.1161/01.cir.75.5.1083

[b34] Romson J. L. . Reduction of the extent of ischemic myocardial injury by neutrophil depletion in the dog. Circulation 67, 1016–1023 (1983). 6831665 10.1161/01.cir.67.5.1016

[b35] Jolly S. R. . Reduction of myocardial infarct size by neutrophil depletion: effect of duration of occlusion. Am. Heart J. 112, 682–690 (1986). 3766367 10.1016/0002-8703(86)90461-8

[b36] Baxter G. F. The neutrophil as a mediator of myocardial ischemia-reperfusion injury: time to move on. Basic Res. Cardiol. 97, 268–275 (2002). 12111036 10.1007/s00395-002-0366-7

[b37] Daley J. M., Thomay A. A., Connolly M. D., Reichner J. S. & Albina J. E. Use of Ly6G-specific monoclonal antibody to deplete neutrophils in mice. J. Leukoc. Biol. 83, 64–70 (2008). 17884993 10.1189/jlb.0407247

[b38] Leuschner F. . Angiotensin-converting enzyme inhibition prevents the release of monocytes from their splenic reservoir in mice with myocardial infarction. Circ. Res. 107, 1364–1373 (2010). 20930148 10.1161/CIRCRESAHA.110.227454 PMC2992104

[b39] Furze R. C. & Rankin S. M. Neutrophil mobilization and clearance in the bone marrow. Immunology 125, 281–288 (2008). 19128361 10.1111/j.1365-2567.2008.02950.x PMC2669132

[b40] Ziegler-Heitbrock L. Monocyte subsets in man and other species. Cellular Immunology 289, 135–139 (2014). 24791698 10.1016/j.cellimm.2014.03.019

[b41] Pons S. . Effects of angiotensin II type 1 receptor blockade in ApoE-deficient mice with post-ischemic heart failure. J. Cardiovasc. Pharmacol. 42, 17–23 (2003). 12827021 10.1097/00005344-200307000-00003

[b42] Vasir J. K. & Labhasetwar V. Biodegradable nanoparticles for cytosolic delivery of therapeutics. Adv. Drug Deliv. Rev. 59, 718–728 (2007). 17683826 10.1016/j.addr.2007.06.003 PMC2002520

[b43] Danhier F. . PLGA-based nanoparticles: An overview of biomedical applications. J. Control Release 161, 505–522 (2012). 22353619 10.1016/j.jconrel.2012.01.043

[b44] Kubo M. . Therapeutic neovascularization by nanotechnology-mediated cell-selective delivery of pitavastatin into the vascular endothelium. Arterioscler. Thromb. Vasc. Biol. 29, 796–801 (2009). 19325146 10.1161/ATVBAHA.108.182584

[b45] Nakashiro S. . Pioglitazone-Incorporated Nanoparticles Prevent Plaque Destabilization and Rupture by Regulating Monocyte/Macrophage Differentiation in ApoE−/− Mice. Arterioscler. Thromb. Vasc. Biol. 36, 491–500 (2016). 26821947 10.1161/ATVBAHA.115.307057

[b46] Guo D. F., Sun Y. L., Hamet P. & Inagami T. The angiotensin II type 1 receptor and receptor-associated proteins. Cell research 11, 165–180 (2001). 11642401 10.1038/sj.cr.7290083

[b47] Zheng Y., Gardner S. E. & Clarke M. C. H. Cell death, damage-associated molecular patterns, and sterile inflammation in cardiovascular disease. Arterioscler. Thromb. Vasc. Biol. 31, 2781–2786 (2011). 22096097 10.1161/ATVBAHA.111.224907

[b48] Jalowy A., Schulz R., Dörge H., Behrends M. & Heusch G. Infarct size reduction by AT1-receptor blockade through a signal cascade of AT2-receptor activation, bradykinin and prostaglandins in pigs. J. Am. Coll. Cardiol. 32, 1787–1796 (1998). 9822110 10.1016/s0735-1097(98)00441-0

[b49] Harada K., Sugaya T., Murakami K., Yazaki Y. & Komuro I. Angiotensin II type 1A receptor knockout mice display less left ventricular remodeling and improved survival after myocardial infarction. Circulation 100, 2093–2099 (1999). 10562266 10.1161/01.cir.100.20.2093

[b50] Ito H. No-reflow phenomenon and prognosis in patients with acute myocardial infarction. Nat. Clin. Pract. Cardiovasc. Med. 3, 499–506 (2006). 16932767 10.1038/ncpcardio0632

[b51] Schwartz Longacre L. . New horizons in cardioprotection: recommendations from the 2010 National Heart, Lung, and Blood Institute Workshop. Circulation 124, 1172–1179 (2011). 21900096 10.1161/CIRCULATIONAHA.111.032698 PMC3709973

[b52] Sugaya T. . Angiotensin II type 1a receptor-deficient mice with hypotension and hyperreninemia. J. Biol. Chem. 270, 18719–18722 (1995). 7642517 10.1074/jbc.270.32.18719

[b53] Ishibashi M. . Bone Marrow-Derived Monocyte Chemoattractant Protein-1 Receptor CCR2 Is Critical in Angiotensin II-Induced Acceleration of Atherosclerosis and Aneurysm Formation in Hypercholesterolemic Mice. Arterioscler. Thromb. Vasc. Biol. 24, e174–e178 (2004). 15331433 10.1161/01.ATV.0000143384.69170.2d

[b54] Matsui Y. . Distinct Roles of Autophagy in the Heart During Ischemia and Reperfusion: Roles of AMP-Activated Protein Kinase and Beclin 1 in Mediating Autophagy. Circ. Res. 100, 914–922 (2007). 17332429 10.1161/01.RES.0000261924.76669.36

[b55] Hsu C. P. . Silent Information Regulator 1 Protects the Heart From Ischemia/Reperfusion. Circulation 122, 2170–2182 (2010). 21060073 10.1161/CIRCULATIONAHA.110.958033 PMC3003297

[b56] Kawashima Y., Yamamoto H., Takeuchi H., Hino T. & Niwa T. Properties of a peptide containing DL-lactide/glycolide copolymer nanospheres prepared by novel emulsion solvent diffusion methods. Eur. J. Pharm. Biopharm. 45, 41–48 (1998). 9689534 10.1016/S0939-6411(97)00121-5

[b57] Takagi H. . Activation of PKN mediates survival of cardiac myocytes in the heart during ischemia/reperfusion. Circ. Res. 107, 642–649 (2010). 20595653 10.1161/CIRCRESAHA.110.217554 PMC3081566

[b58] Han K. H. . HMG-CoA reductase inhibition reduces monocyte CC chemokine receptor 2 expression and monocyte chemoattractant protein-1-mediated monocyte recruitment *in vivo*. Circulation 111, 1439–1447 (2005). 15781755 10.1161/01.CIR.0000158484.18024.1F

