# Supplementary material for: Nanoparticle-Mediated Delivery of Irbesartan Induces Cardioprotection from Myocardial Ischemia-Reperfusion Injury by Antagonizing Monocyte-Mediated Inflammation
Source: Sci Rep. 2016 Jul 11;6:29601. doi: 10.1038/srep29601 (PMC4939605; doi:10.1038/srep29601)
Supplement: Supplementary Information [file srep29601-s1.pdf]

Supplementary Information

Nanoparticle-Mediated Delivery of Irbesartan Induces Cardioprotection from Myocardial Ischemia-Reperfusion Injury by Antagonizing Monocyte-Mediated Inflammation

Yasuhiro Nakano, MD; Tetsuya Matoba, MD, PhD; Masaki Tokutome, MD; Daiki Funamoto, PhD; Shunsuke Katsuki, MD; Gentaro Ikeda, MD; Kazuhiro Nagaoka, MD; Ayako Ishikita, MD; Kaku Nakano, PhD; Jun-ichiro Koga MD, PhD; Kenji Sunagawa, MD, PhD; Kensuke Egashira, MD, PhD

Supplemental Methods

Mouse myocardial IR injury model

Adult male C57BL/6J mice (9-13 weeks old) (CLEA Japan, Inc), AT1R-defecient mice on C57BL/6J background (gift from Dr. Hiroyuki Yamada, Kyoto Prefectural University School of Medicine)[1] and CCR2$^{-/-}$ mice on C57BL/6J and 129/svjae hybrids background[2] were used in this study. All experiments were reviewed and approved by the committee on ethics on animal experiments, Kyushu University Faculty of Medicine, and were conducted according to the guideline of the American Physiological Society. Based on our previous animal studies, we estimated to use at least 6 animals per condition. The number of mice proposed was needed to ensure that experimental derived data achieve statistical significance. The pre-specified exclusion criteria were the body weight of 25g or less and prematurely death after IR. The myocardial IR model was based on previously described methods[3,4]. After anesthesia with an intraperitoneal injection of pentobarbital sodium (50 mg kg^{-1}), the mice were intubated and ventilated with a respirator. The heart was exposed by a left thoracotomy on heating pads. Myocardial ischemia was produced by ligating the anterior descending branch of the left coronary artery (LAD) using an 8-0 nylon suture, with silicon tubing (1 mm OD) placed on top of the LAD, 2 mm below the border between left atrium and LV. After occlusion for 30 min, the silicon tubing was removed to achieve reperfusion. The mice were kept warm by a heating pad until they were completely awake.

Experimental protocol

Experimental protocol 1: To examine the response of leukocytes within the injured myocardium after IR, animals were subjected to myocardial ischemia for 30 minutes, followed by coronary reperfusion. After 3, 6, 12, 24, and 48 hours of reperfusion, mice were

sacrificed, and their hearts were harvested. Flow cytometric analysis was then performed.

Experimental protocol 2: To examine the role of neutrophil-mediated inflammation in myocardial IR injury, WT mice were intraperitoneally injected with anti-Ly6G 1A8 monoclonal antibody (100 μg body^{-1}, BioXCell, West Lebanon, NH) 24 hours prior to the induction of myocardial IR. To examine the role of monocyte-mediated inflammation in myocardial IR injury, CCR2$^{-/-}$ mice were subjected to myocardial IR. Twelve hours after reperfusion, animals were sacrificed, and flow cytometric analysis was performed. The infarct size was measured after 3, 12, 24 hours of reperfusion.

Experimental protocol 3: To examine the biodistribution of ICG-NP, animals were intravenously injected with ICG-NP, sacrificed 5 minutes after injection and perfused with cold phosphate-buffered saline (PBS). Then the heart, spleen, liver and kidneys were harvested and imaged by fluorescence reflectance imaging using FMT-2000's planar imaging capability.

Experimental protocol 4: To examine the distribution of FITC-NP, animals were divided into 3 groups that received intravenous injection of following drugs: 1) vehicle (saline 200 μL body^{-1}), 2) FITC alone (FITC 81.2 μg in saline 200 μL body^{-1}), 3) FITC-NP (PLGA 2 mg containing 81.2 μg FITC in saline 200 μL body^{-1}). Animals were sacrificed and mice were perfused with cold PBS. Histopathological analysis was performed after 3 hours of reperfusion and flow cytometric analysis was performed after 6 hours of reperfusion.

Experiment protocol 5: To examine the plasma and tissue concentrations of irbesartan, animals were divided the two groups and sacrificed 30 minutes, 3 hours and 24 hours after reperfusion. Peripheral blood was drawn by cardiac puncture and mice were perfused with cold PBS. Then heart and spleen were harvested.

Experimental protocol 6: To examine the effects of therapeutic agents on infarct size after IR, animals were divided the following groups: 1) vehicle (0.5% carboxymethylcellulose 200 μL body^{-1} day^{-1}, 7 days, oral); 2) irbesartan (50 mg kg^{-1} irbesartan in carboxymethylcellulose 200 μL body^{-1} day^{-1}, 7 days, oral); 3) vehicle (saline 200 μL body^{-1}, iv) in WT mice and AT1R deficient mice; 4) FITC-NP (FITC 81.2 μg in saline 200 μL body^{-1}, iv); 5) irbesartan alone (3.0 mg kg^{-1} in saline 200 μL body^{-1}, iv); and 6) irbesartan-NP (PLGA containing 3.0 mg kg^{-1} irbesartan in saline 200 μL body^{-1}, iv). In another set of experiments, GW9662 (2 mg kg^{-1}; Sigma Aldrich), a PPARγ inhibitor, was intravenously administered 1 hour before LAD ligation. Echocardiography was performed using a Vevo 2100 ultrasound system at baseline, 1 week, 2 weeks and 3 weeks after reperfusion. Measurement of systolic blood pressure and heart rate by using tail-cuff method were performed at baseline, 6 hours and 24 hours after reperfusion.

Experimental protocol 7: To examine the impact of the reducing Ly6C^{high} monocytes on infarct size reduction by irbesartan-NP, CCR2$^{-/-}$ mice were divided into 2 groups receiving the following drugs: 1) vehicle (saline 200 μL body^{-1}); and 2) irbesartan-NP (PLGA containing 3.0 mg kg^{-1} irbesartan in saline 200 μL body^{-1}). Twenty-hour hours after reperfusion, animals were sacrificed, and the infarct sizes were measured.

Experimental protocol 8: To examine the effects of irbesartan-NP on cytochrome c leakage from mitochondria into the cytosol after reperfusion, animals were divided into 3 groups receiving the following drugs: 1) vehicle (saline 200 μL body^{-1}); 2) vehicle (saline 200 μL body^{-1}) after pretreatment with cyclosporine A (10 mg kg^{-1}) every 12 hours starting 36 hours before ischemia; and 3) irbesartan-NP (PLGA containing 3.0 mg kg^{-1} irbesartan in saline 200 μL body^{-1}). The treatment experimental protocols were performed after randomization.

Multiple observers blinded to all the experimental protocols performed the quantitative analysis.

Quantification of myocardial infarction size

Twenty-four hours after reperfusion, the mice were anesthetized with an intraperitoneal injection of pentobarbital sodium and were intubated. The LAD was re-occluded, and 2% Evans blue dye (Sigma Aldrich) was injected via the inferior vena cava to identify the area at risk (AAR). The heart was then excised and perfused with saline and cut into sequential 1-mm-thick cross-sections. The sections were incubated with 1% 2,3,5-triphenyltetrazolium chloride (TTC, Sigma Aldrich) for 10 minutes at 37 °C and were photographed with a stereomicroscope (Nikon, HC-2500). The MI area (TTC negative, white), non-MI area within the AAR (TTC positive/Evans blue negative, red), non-ischemic area (TTC positive/Evans blue positive, purple), and AAR (Evans blue negative) were analyzed using Adobe Photoshop 6.0 (Adobe Systems, San Jose, CA, USA) and Scion Image 1.62 for Windows (Scion, Frederick, MD, USA).

Preparation of PLGA nanoparticles

PLGA with an average molecular weight of 20,000 and a copolymer ratio of lactide to glycolide of 75:25 (Wako Pure Chemical Industries Ltd, Osaka, Japan) was used as a matrix for the nanoparticles, whereas polyvinyl alcohol (PVA-403; Kuraray, Osaka, Japan) was used as a dispersing agent. PLGA nanoparticles encapsulated with fluorescein isothiocyanate (FITC; Dojin Chemical, Tokyo, Japan) (FITC-NP), indocyanine green (ICG; Tokyo Chemical Industry Co. Ltd., Tokyo, Japan) (ICG-NP) and irbesartan (Shionogi & Co. Ltd., Osaka, Japan) (irbesartan-NP) were prepared using an emulsion solvent diffusion method, as previously reported[5,6]. PLGA (500 mg) and irbesartan (each concentration) were dissolved in

30-mL acetone. The resultant organic solution was poured into 60 mL of an aqueous PVA solution (0.5% or 2% w/v in distilled water) and stirred at 400 rpm, 40 ºC during the operation. The dispersed solution was then centrifuged (20,000 g for 20 minutes) and the sediments were resuspended in distilled water. The process was repeated and the resultant dispersion was freeze-dried. The redispersion of the nanoparticles prepared in the 2% PVA condition was better than those prepared in 0.5% PVA condition after freeze-drying. It was assumed that PVA on the surface of nanoparticle might improve the wettability and redispersibility[7]. Therefore, we selected the 2% PVA concentration in aqueous phase to prepare irbesartan-PLGA nanoparticles in this system (Supplementary Table 4).

Physicochemical characteristic of the irbesartan-nanoparticles

In this study, drug concentration in the organic phase and PVA concentration in aqueous phase were examined to control the particle size and drug content of irbesartan-NP. A transmission electron microscope (TEM, Hitachi H7000E, Tokyo, Japan) was used for the morphological examination of irbesartan-NP (Supplementary Fig. S8A, B). The particle size was analyzed by a dynamic light scattering method (Microtrack UPA150, Nikkiso, Tokyo, Japan)[8] (Supplementary Fig. S8C). A sample of nanoparticle suspension in distilled water was used for particle size analysis. The average diameter of nanoparticles was approximately 200 nm in the all experimental condition (Supplementary Table 4). The size distribution was also similar between each drug concentration in organic phase (Supplementary Fig. S8C). The particle size of FITC-NP and ICG-NP was analyzed by the same method, the diameter of FITC-NP was 225 nm and ICG-NP was 253 nm, respectively. On the other hand, increasing of the drug concentration in the organic phase led to a gradual increase of drug content in the nanoparticles (Supplementary Table 4). The drug content was analyzed by high-performance

liquid chromatography (Waters Alliance e2695, Waters Corporations, Milford, MA, USA) followed by ultraviolet (UV) detection. The drug content in nanoparticle were calculated from the equation: drug content (%)=weight of recovered drug in nanoparticles/weight of nanoparticles. The irbesartan-NP contained 3.29% (wt vol^{-1}) irbesartan. The drug content of FITC-NP and ICG-NP was calculated by the same method; the FITC-NP contained 4.06% (wt vol^{-1}) FITC and ICG-NP contained 0.16%(wt vol^{-1}) ICG. Surface charge (zeta potential) was also analyzed by Zetasizer Nano (Sysmex, Hyogo, Japan) and was anionic [-20.3 mV (FITC-NP), -24.0 mV (ICG) and -18.4 mV (irbesartan-NP)].

The distribution of FITC in the heart and peripheral blood leukocytes was examined. After myocardial IR, animals were sacrificed, and peripheral blood was drawn by means of cardiac puncture with EDTA (ethylenediaminetetraacetic acid) as an anticoagulant, and red cells were lysed with VersaLyse Lysing Solution (Becton Dickinson Biosciences, San Jose, CA, USA).

Distribution of FITC-nanoparticles

The distribution of FITC in the heart and peripheral blood leukocytes was examined. After myocardial IR, animals were sacrificed, and peripheral blood was drawn by means of cardiac puncture with EDTA (ethylenediaminetetraacetic acid) as an anticoagulant, and red cells were lysed with VersaLyse Lysing Solution (Becton Dickinson Biosciences, San Jose, CA, USA). The distribution of FITC-NP in the peripheral blood leukocytes was analyzed with a FACSCalibur cytometer (Becton Dickinson Biosciences). The heart was harvested and fixed in 10% phosphate-buffered formalin (pH 7.4), and the distribution of FITC-NP was analyzed in 5-µm OCT-embedded sections. Sections of the heart were evaluated by fluorescence microscopy.

Flow cytometry

Peripheral blood was drawn via a cardiac puncture, and red blood cells were lysed with VersaLyse Lysing solution for 10 minutes at room temperature. Hearts were removed and digested with a cocktail of 450 U mL^{-1} collagenase type I, 125 U mL^{-1} collagenase type XI, 60 U mL^{-1} DNase I and 60 U mL^{-1} hyaluronidase (all enzymes were obtained from Sigma-Aldrich) in PBS containing 20 mM Hepes at 37 °C for 1 h. The cell suspension was centrifuged at 300 x g for 5 minutes at 4 °C. After blocking the Fc receptor with anti-CD16/32 mAb (BD Pharmingen, San Diego, California) for 5 minutes at 4 °C, cell suspensions were incubated with a cocktail of mAb against lineage markers [T cells (CD90-PE, 53-2.1), B cells (B220-PE, RA3-6B2), NK cells (CD49b-PE, DX5 and NK1.1-PE, PK136), and granulocytes (Ly6G-PE, 1A8)], myeloid cells (CD11b-APC, M1/70), monocyte subsets (Ly6C-FITC, -APC-Cy7, AL-21) (BD Pharmingen, San Diego, CA. USA), and macrophage (F4/80-FITC, Cl:A3-1) (AbD Serotec, Kidlington, UK) for 1 h at 4 °C; all leukocytes were then analyzed with FACS Calibur (BD Biosciences, San Jose, CA, USA). The leukocytes were also incubated with appropriate isotype controls (BD Pharmingen, San Diego, CA, USA).

Monocytes/macrophages were identified as $CD11b^{high}(CD90/B220/CD49b/NK1.1/Ly6G)^{low}Ly6C^{high/low}$, macrophages were identified as $CD11b^{high}(CD90/B220/CD49b/NK1.1/Ly6G)^{low}Ly6C^{low}F4/80^{high}$, neutrophils were identified as $CD11b^{high}(CD90/B220/CD49b/NK1.1/Ly6G)^{high}$, and lymphocytes were identified as $CD11b^{low}(CD90/B220/CD49b/NK1.1/Ly6G)^{high}$ as previously described[9].

Distribution of FITC-nanoparticles

The distribution of FITC in the heart and peripheral blood leukocytes was examined. After

myocardial IR, animals were sacrificed, and peripheral blood was drawn by means of cardiac puncture with EDTA (ethylenediaminetetraacetic acid) as an anticoagulant, and red cells were lysed with VersaLyse Lysing Solution (Becton Dickinson Biosciences, San Jose, CA, USA). The distribution of FITC-NP in the peripheral blood leukocytes was analyzed with a FACSCalibur cytometer (Becton Dickinson Biosciences). The heart was harvested and fixed in 10% phosphate-buffered formalin (pH 7.4), and the distribution of FITC-NP was analyzed in 5-µm OCT-embedded sections. Sections of the heart were evaluated by fluorescence microscopy.

Histological and immunohistochemical analyses

Twelve hours after reperfusion, hearts were harvested and fixed overnight in 10% buffered-formalin. After fixation, the tissue was embedded in paraffin. Serial cross-sections (5-µm thick) were used for analysis. The sections were subjected to immunohistology using anti-MCP-1 antibodies (1:200, Santa Cruz Biotechnology). The degree of MCP-1 expression in the AAR 12 hours after reperfusion was evaluated. Digital images of 5 fields in the AAR per heart were stored, and the MCP-1 staining area was assessed using a 20 × objective.

Western blot analysis

Homogenates of IR myocardium were analyzed with immunoblotting. At predetermined time points, ischemic myocardium was isolated and analyzed as previously reported[10]. Briefly, frozen samples were homogenized in lysis buffer, and proteins were separated on SDS-polyacrylamide gels and then blotted to PVDF membranes. The following antibodies were used as primary antibodies: cytochrome c (cytosol fraction; 1:1000, Santa Cruz Biotechnology), VDAC (1:1000, Cell Signaling), and GAPDH (1:2500, Santa Cruz Biotechnology).

PPARγ and NF-κB activity in myocardium

Nuclear extracts were prepared from the myocardium homogenates using a nuclear extract kit (NE-PER Nuclear and Cytoplasmic Extraction Reagents; Thermo Fisher Scientific Inc., Rockford, IL) according to the manufacturer's instructions. The protein was measured using a BCA Protein Assay kit (Thermo Fisher Scientific Inc.). PPARγ and NF-κB activation were assayed using an ELISA-based PPARγ activation TransAM kit (Active Motif, Rixensart, Belgium) and an ELISA-based NF-κB activation TransAM kit (Active Motif, Rixensart, Belgium), which were used according to the manufacturer's instructions. Briefly, nuclear protein samples were incubated for 1 hour in a 96-well plate coated with an oligonucleotide that contains the PPAR response element domain (5′-AACTAGGTCAAAGGTCA-3′) or the NF-κB consensus sequence (5′-GGGACTTTCC-3′). After washing, PPARγ antibody (1:1,000 dilution) or NF-κB antibody (1:1,000 dilution) was added to these wells and incubated for 1 hour. After incubation for 1 hour with a secondary horseradish peroxidase–conjugated antibody (1:1,000 dilution), specific binding was detected by colorimetric estimation at 450 nm with a reference wavelength of 655 nm.

Isolation of mouse myocardial mitochondria

Mice heart mitochondria were isolated according to the manufacturer's protocol (Abcam)[11]. One hour after IR, mice were anesthetized, and the hearts were quickly excised. The IR myocardium was isolated, minced on ice, resuspended in isolation buffer and homogenized with a glass Dounce homogenizer and Teflon pestle. Homogenates were centrifuged at 1,000 × *g* for 5 minutes at 4 ºC. The supernatant was re-centrifuged at 12,000 × *g* for 15 minutes to pellet the mitochondria twice. The pellets were collected, resuspended and frozen at -80 ºC.

Echocardiography

Mice were anesthetized with inhaled isoflurane (1-2%; Abbott) and placed in supine position on a warming pad. Echocardiographic measurements were performed at baseline and 1-week, 2-week and 3-week after reperfusion. Short axis values of left ventricular end systolic and end diastolic dimension were obtained using a Vevo 2100 ultrasound system (Primetech Inc).

Fluorescence molecular tomography (FMT) and Fluorescence reflectance imaging (FRI)

Five nanomoles of pan-cathepsin protease sensor (ProSence 680, PerkinElmer) was intravenously administered after 24-hour of reperfusion. Twenty-four hours later, the animals were anesthetized with an intraperitoneal injection of pentobarbital sodium and were scanned with FMT-2000 system (PerkinElmer). The volume of interest (ROI) for heart was placed by reference to previous report[12]. After animals were imaged *in vivo*, they were sacrificed and the heart was removed. The heart was then cut into sequential 1-mm-thick cross-sections and imaged by fluorescence reflectance imaging using FMT-2000's planar imaging capability. In the experimental protocol of distribution of ICG-NP, mice were sacrificed after 5 minutes of intravenous administration of ICG-NP. The mice were then perfused with cold PBS, and the organs (heart, lungs, liver, spleen, kidneys) were removed and imaged by FRI.

Langendorff perfusion and infarct model.

Mice were anesthetized with an intraperitoneal injection of pentobarbital sodium, and the heart was rapidly excised and arrested in icecold buffer. The aorta was then cannulated and retrogradely perfused at constant pressure of 80 mmHg with 37 °C Krebs–Heinseleit (KH) buffer. Hearts were Langendorff-perfused with KH buffer for 20 min (equilibration) before being subjected to 45 min of no-flow ischemia. Individual five hearts were treated with either vehicle (KH buffer) or irbesartan-NP (25 μM) for 30 minutes after ischemia and during the entire 120 min reperfusion. At the end of the 120-min reflow, hearts were snap-frozen in

liquid nitrogen and were sliced into sequential 1-mm-thick cross-sections. The sections were incubated with 2% 2,3,5-triphenyltetrazolium chloride (TTC, Sigma Aldrich) for 10 minutes at 37 °C and were photographed with a stereomicroscope (Nikon, HC-2500)[13,14].

In vitro drug release kinetics

Irbesartan-NP was dissolved in PBS (10 mM, pH 7.4) (0.1 mg mL^{-1}) and loaded to 10 k MWCO Slide-a-lyzer dialysis cassettes (0.1–0.5 mL, Thermo Scientific) and dialyzed against 1000 mL PBS (10 mM, pH 7.4) at 37 °C. At each time point, 50 µL were removed from inside of the cassette for the measurement of remaining irbesartan[15].

Chemotaxis assay

THP-1, a human monocyte cell line, was obtained from the German Collection of Microorganisms and Cell Cultures (DSMZ; Brauschweig, Germany). The cells were cultured in RPMI 1640 with 10% FBS at 37 °C in a 5% CO_2 environment until they were subconfluent. The growth medium was replaced with starvation medium with irbesartan-NP containing 0.1303 to 13.03 µg mL^{-1} of PLGA and 0.01 to 1.0 µM of irbesartan, FITC-NP containing 0.1303 to 13.03 µg mL^{-1} of PLGA, or vehicle alone for 24 hours. The chemotactic activity of THP-1 cells in response to 10 ng mL^{-1} MCP-1 was measured in a 96-well microchemotaxis Boyden chamber (ChemoTx; Neuroprobe), as described previously[16]. Monocytes that had transmigrated through the micropore were stained with trypan blue. The number of monocytes that migrated in response to MCP-1 was counted.

Measurements of irbesartan concentrations in the plasma and tissues

Irbesartan concentrations in the plasma and tissues were measured at predetermined time points by liquid chromatography coupled to tandem mass spectrometry (LC/MS/MS). Briefly, the high-performance liquid chromatography (HPLC) analysis was performed using the

Waters UPLC system (Waters Corporation, Milford, MA, USA). The column temperature was maintained at 40 ºC. The flow rate was 0.75 mL min^{-1}. Plasma or tissue homogenate samples were precipitated using acetonitrile, and the supernatant solutions were injected from the autosampler into the HPLC system. The mass spectrometers used in this study were Quattro Ultima Pt (Waters Corporation, Milford, MA, USA) and API5000 (AB SCIEX, Framingham, MA, USA) in the case of lower concentrations. The electrospray interface was operated in the positive ion mode at 4500 V, and the temperature was optimized for each instruments. The analytical data were processed using MassLynx (version 4.0, Waters Corporation, Milford, MA, USA) or Analyst (version 1.4.2, AB SCIEX, Framingham, MA, USA).

Supplemental Figures and Figure Legends

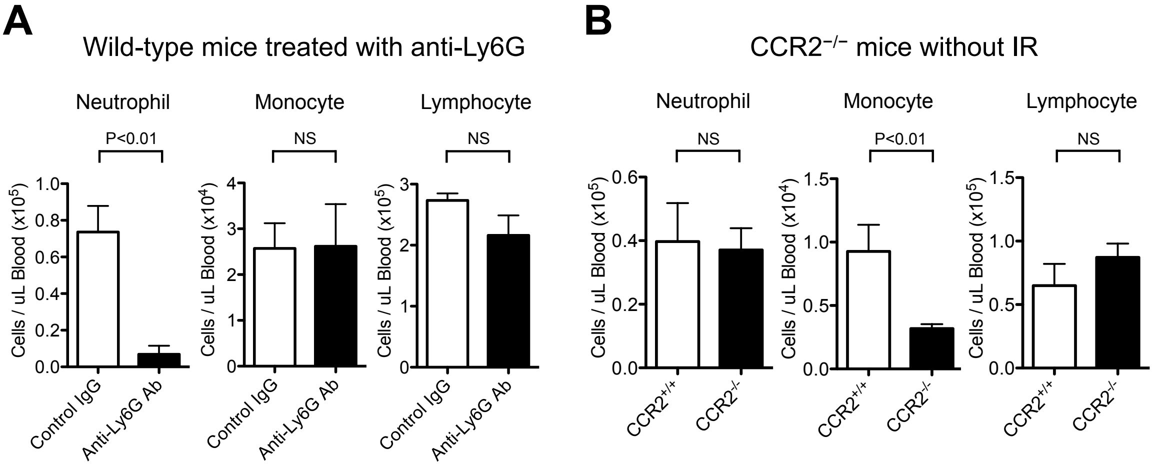

Supplementary Figure 1.

(A) The effects of the anti-Ly6G 1A8 monoclonal antibody on neutrophils, monocytes and lymphocytes in the blood of normal mice. Data are expressed as the mean ± SD (N=3 per group). Data are compared using the unpaired *t*-test. (B) The number of neutrophils, monocytes and lymphocytes in the blood of $CCR2^{-/-}$ mice without IR. Data are expressed as the mean ± SD (N=5 per group). Data are compared using the unpaired *t*-test.

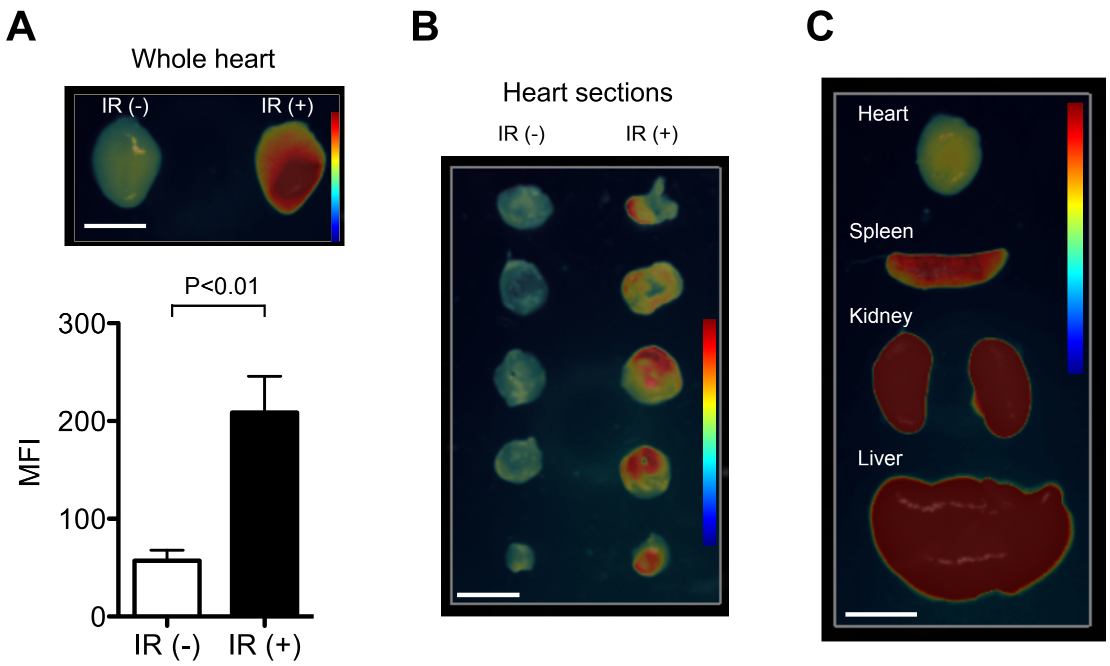

Supplementary Figure 2.

(A) *Ex vivo* FRI imaging of the whole heart in sham operated mouse or myocardial IR mouse after 5 minutes of intravenous administration of ICG-NP. Quantification of the ICG mean fluorescence intensity (MFI) in the heart. Data shown as mean ± SD (N=5 per group). Data are compared using the unpaired *t*-test. (B) *Ex vivo* FRI imaging of the sections of heart in sham operated mouse or myocardial IR mouse after 5 minutes of intravenous administration of ICG-NP. (C) *Ex vivo* FRI imaging of the heart, spleen, kidneys and liver after 5 minutes of intravenous administration of ICG-NP. Scale bar: 10mm. FRI: fluorescence reflectance imaging, ICG: indocyanine green, IR: ischemia reperfusion.

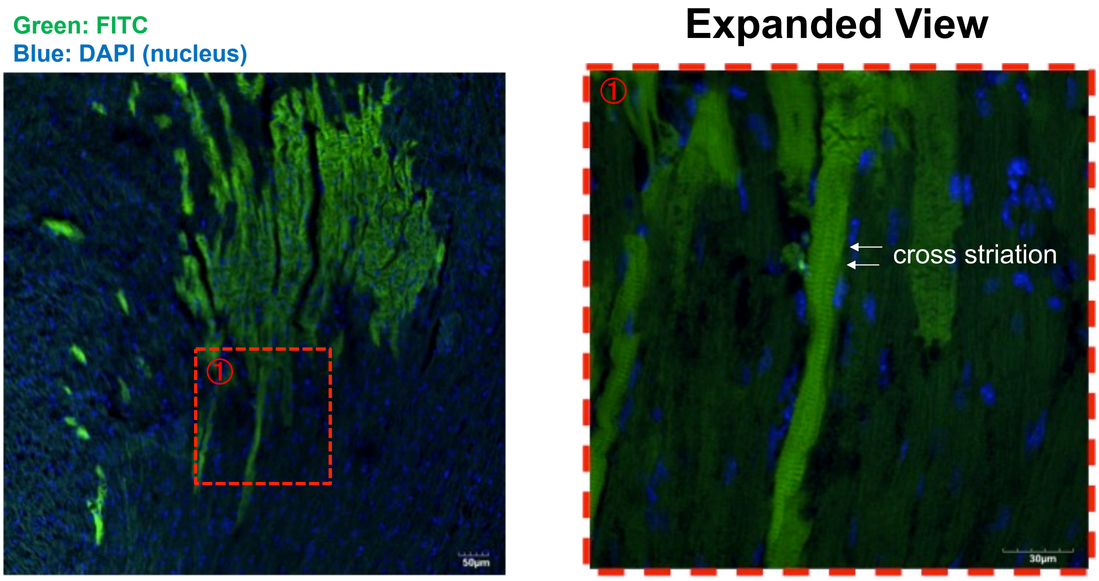

Supplementary Figure 3.

Representative fluorescence photomicrographs of cross-sections from hearts treated with FITC-NP. Green indicates FITC, and blue indicates nuclei (DAPI). FITC signals were detected in cardiomyocytes.

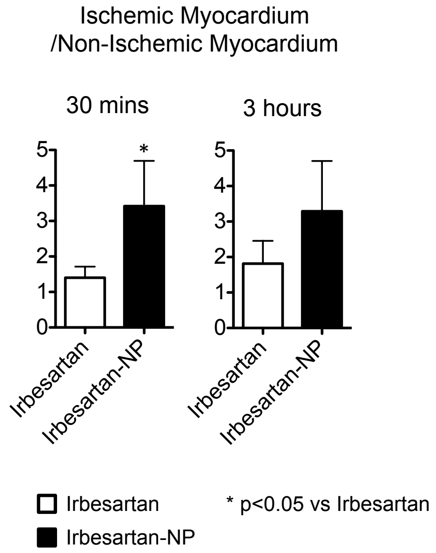

Supplementary Figure 4.

Irbesartan concentrations in ischemic over non-ischemic myocardium after 30 minutes and 3 hours of reperfusion. Data are expressed as the mean ± SD (N=4-5 per group). Data are compared using the unpaired *t*-test.

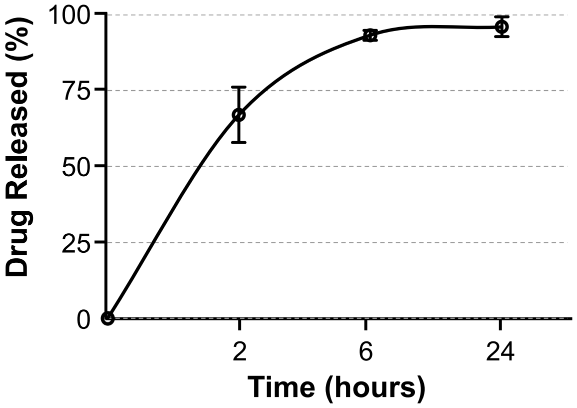

Supplementary Figure 5.

In vitro time course of cumulative irbesartan release from the irbesartan-NP (N=3 each). The percentage of incremental quantities of released FITC was plotted against time. Data are expressed as the mean ± SD (N=3 per group).

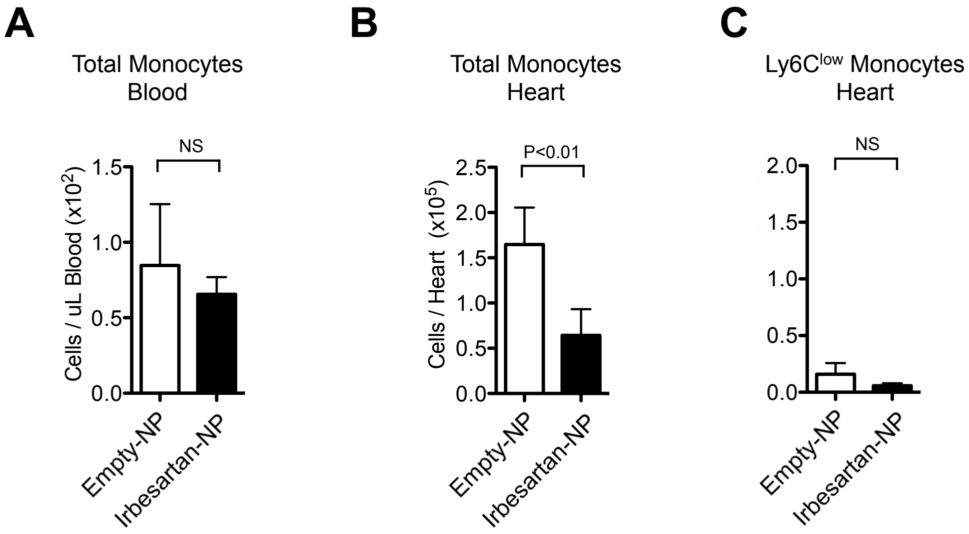

Supplementary Figure 6.

(A), (B) The number of total monocytes in the peripheral blood and the IR hearts after 12 hours of reperfusion. (C) The number of Ly6C^{low} monocytes in the IR hearts after 12 hours of reperfusion. Data are expressed as the mean ±SD (N=5 per group). Data are compared using the unpaired *t*-test.

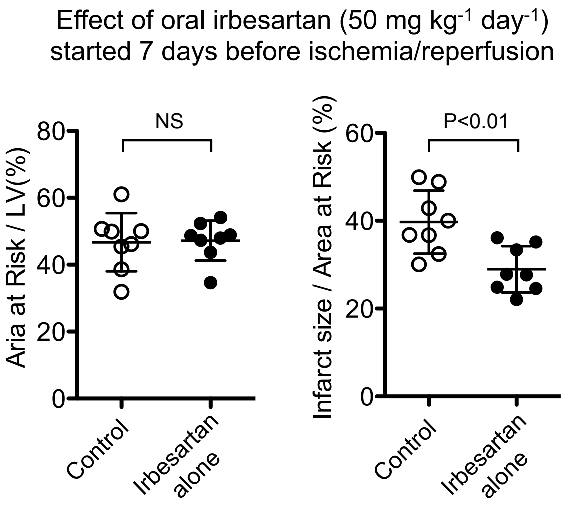

Supplementary Figure 7.

The effects of pretreatment with irbesartan alone (50 mg kg^{-1} day^{-1}, 7 days, oral) in an IR mice model. Data are expressed as the mean ± SD (N=8 per group). Data are compared using one-way ANOVA followed by Bonferroni's multiple comparison tests. LV: left ventricle.

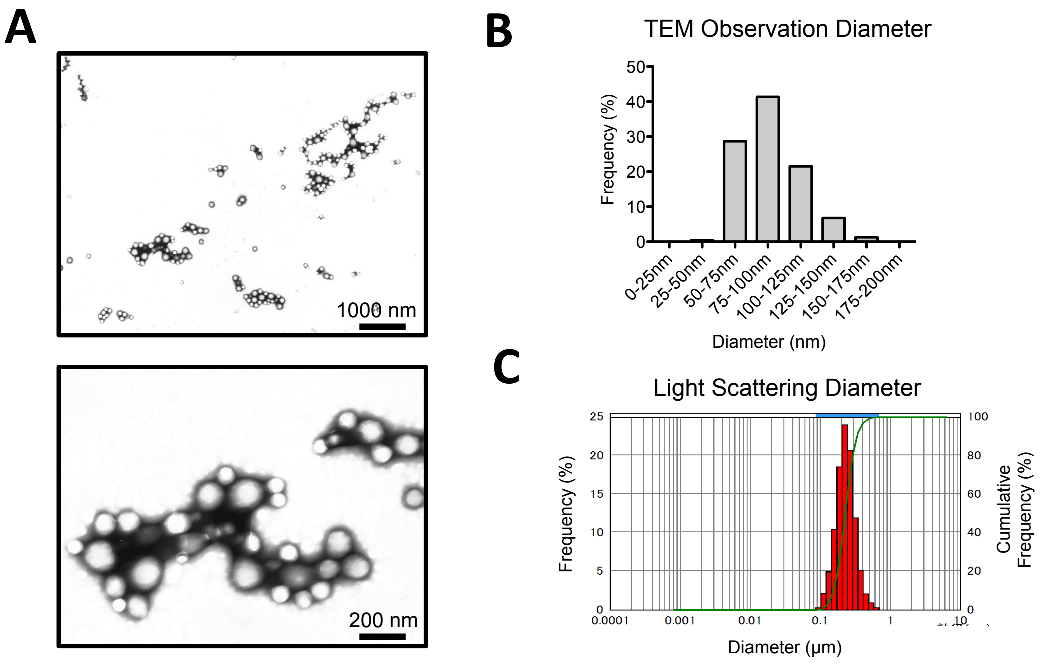

Supplementary Figure 8.

(A) Transmission electron microscopy photograph of irbesartan-NP. (B) Particle size distributions of irbesartan-NP. (C) Diameter of irbesartan-NP by dynamic light scattering.

Supplementary Table 1. Effects of irbesartan-NP on hemodynamic parameters

	Baseline	6 hours after IR	24 hours after IR
**SBP (mmHg)**			
Saline	110.9±3.9	98.3±2.3	105.1±2.5
Irbesartan solution	107.2±4.2	102.0±2.6	110.6±1.7
Irbesartan-NP	107.1±3.2	96.4±3.1	101.8±2.4
**HR (bpm)**			
Saline	599.1±6.5	584.4±19.7	606.3±22.8
Irbesartan solution	598.7±11.8	594.3±8.1	608.5±10.6
Irbesartan-NP	594.6±9.7	561.0±21.5	562.1±16.9

SBP: Systolic blood pressure, HR: heart rate

Data are expressed at the mean ± SEM (n=5 each).

Supplementary Table 2. Effects of irbesartan-NP on cardiac remodeling measured by echocardiography

Groups		Weeks after IR			
		Baseline	1 week	2 weeks	3 weeks
Saline	LVEDD (mm)	3.51 ± 0.17	4.08 ± 0.21	4.32 ± 0.14	4.54 ± 0.22
	LVEDS (mm)	2.16 ± 0.13	3.27 ± 0.27	3.59 ± 0.15	3.76 ± 0.26
	LVEF (%)	69.7 ± 3.29	41.0 ± 4.88	35.7 ± 4.40	36.0 ± 4.38
	LVFS (%)	38.5 ± 2.66	19.8 ± 2.66	17.0 ± 2.38	17.2 ± 2.32
Irbesartan solution (3.0 mg/kg)	LVEDD (mm)	3.51 ± 0.21	4.11 ± 0.17	4.26 ± 0.17	4.43 ± 0.19
	LVEDS (mm)	2.19 ± 0.20	3.28 ± 0.22	3.50 ± 0.22	3.66 ± 0.19
	LVEF (%)	68.7 ± 4.25	41.8 ± 4.56	37.04 ± 4.61	36.3 ± 3.90
	LVFS (%)	37.7 ± 3.44	20.3 ± 2.53	17.7 ± 2.48	17.4 ± 2.12
Irbesartan-NP (containing 3.0 mg/kg irbesartan)	LVEDD (mm)	3.49 ± 0.18	3.82 ± 0.11*	3.87 ± 0.09***	4.00 ± 0.13***
	LVEDS (mm)	2.09 ± 0.16	2.81 ± 0.14**	2.90 ± 0.15***	2.94 ± 0.16***
	LVEF (%)	71.63 ± 4.10	52.4 ± 2.95***	50.4 ± 4.13***	52.2 ± 3.05***
	LVFS (%)	40.06 ± 3.36	26.4 ± 1.79***	25.2 ± 2.51***	26.4 ± 1.85***

Data are expressed as the mean ± SD (n=7 each). *$P<0.05$, **$P<0.01$, and ***$P<0.001$ versus saline group. Left ventricular dimension end diastolic dimension (LVEDD), left ventricular dimension end systolic dimension (LVEDS), left ventricular ejection fractions (LVEF), left ventricular fractional shortening (LVFS)

Supplementary Table 3. The role of neutrophils on myocardial IR injury

Method of leukocyte removal	Effects on Leukocytes	Infarct size	Animal	Ischemia	Reperfusion	Author
**Leukocyte filter (Imugard IG500)**	Blood: N↓, Mo↓, Ly↓<br>Heart: MPO activity↓	Reduction	Dog	90 min	2 h	Litt[17]
**Anti-PMN serum**	Blood: N↓, M→, Ly→<br>Heart: Leukocytes↓ (HE stain)	Reduction	Dog	90 min	6 h	Romson[18]
**Anti-PMN serum**	Blood: N↓, M→, Ly→	Reduction<br>No difference	Dog	90 min<br>4 h	24 h<br>6 h	Jolly[19]
**Anti-PMN serum**	Blood: N↓, M→, Ly→	No difference	Dog	3 h	21 h	Chatelain[20]
**Anti-PMN serum**	Blood: N↓<br>Heart: CD18 cells↓	Reduction	Rat	30 min	3 h	Granfeldt[21]
**Anti-PMN serum and Mechlorethanime**	Blood: N↓, M↓	Reduction	Dog	2 h	6 h	Lorgeril[22]
**Anti-CD18 antibody**	Heart: MPO activity↓	Reduction	Dog	90 min	48 h	Arai[23]
**Anti-CD18 antibody**	Heart: ^{111}In labeled PMN↓	No difference	Dog	90 min	3 h	Tanaka[24]
**Anti-CD18 antibody**	Blood: N→<br>Heart: MPO activity↓	Reduction<br>No difference	Rabbit	30 min<br>45 min	3 h<br>3 h	Williams[25]
**Anti-CD18 antibody (MAbR15.7)**	Heart: MPO activity↓	Reduction	Cat	90 min	4.5 h	Ma[26]
**Anti-CD11b/CD18 antibody**	Blood: N↓<br>Heart: Leukocyte↓ (HE	Reduction	Dog	90 min	6 h	Simpson[27]

	stain)					
**Anti-CD11b antibody**	Heart: MPO activity→, Leukocyte→ (HE stain)	Reduction	Dog	90 min	72 h	Simpson[28]
**Anti-ICAM-1 antibody (CL18/6)**	Heart: MPO activity↓	Reduction	Dog	60 min	5 h	Hartman[29]
**Anti-ICAM-1 antibody (mAb RR1/1)**	Blood: WBC↓, N↓	Reduction	Rabbit	30 min	2 h	Zhao[30]
**Anti-C5 antibody (18A)**	Heart: MPO activity↓	Reduction	Rat	30 min	4 h	Vakeva[31]
**Anti-P-selectin antibody (PB1.3)**	Heart: MPO activity↓	Reduction	Dog	120 min	4 h	Lefer[32]
**Sialyl Lewis^x Analogue (CY1503)**	Heart: MPO activity→	No difference	Dog	90 min	48 h	Gill[33]
**Sialyl Lewis^x Analogue (GM 1380)**	Blood: N→ Heart: MPO activity↓	Reduction	Rabbit	30 min	5 h	Kilgore[34]
**Sialyl Lewis^x Analogue (CY1503)**	Heart: Leukocyte↓ (HE stain)	No difference	Rabbit	30 min	4 h	Birnbaum[35]
**Sialyl Lewis^x Analogue (CY1503)**	Heart: MPO activity↓	Reduction	Dog	90 min	4.5 h	Lefer[36]
**Sialyl Lewis^x Analogue (CY150[illegible]**	Heart: MPO activity↓, Leukocyte↓ (HE stain)	Reduction	Dog	80 min	1 h	Sliver[37]
**ibuprofen**	Heart: Leukocyte→ (HE stain)	Reduction	Baboon	4 h	24 h	Crawford[38]

**ibuprofen**	Heart: ^{111}In labeled Leukocyte↓	Reduction	Dog	60 min	24 h	Romson[39]
**ibuprofen**	Heart: MPO activity→	No difference	Dog	60 min	2 h	Allan[40]
**Lipoxygenase/cyclooxygenase inhibitor**	Blood: Leukocyte→ Heart: Leukocyte↓ (HE stain)	Reduction	Dog	60 min	5 h	Mullane[41]
**Recombinant soluble P-selectin glycoprotein ligand-1 (rsPSGL-1)**	Heart: MPO activity↓	Reduction	Cat	90 min	3 h	Hayward[42]
**Vascular endothelial cadherin (VEcadherin)-FasL Tg mouse**	Heart: MPO activity↓	Reduction	Mouse	30 min	72 h	Yang[43]

N: neutrophil, M: monocyte, Ly: lymphocyte, MPO: myeloperoxidase, HE: hematoxylin-eosin, PMN: polymorphonuclear leukocyte, ICAM: intracel lular adhesion molecule, In: indium.

Supplementary Table 4. Physicochemical characteristics of the PLGA nanoparticles

Protocol	PVA concentration (%w/v)	PLGA : Drug (mg)	N	Particle size (nm)	Polydispersity index	Drug content (%)
1	0.5	500 : 0	3	201±2.5	0.68±0.13	0
2	0.5	500 : 12.5	3	199±0.75	0.77±0.02	0.8±0.00
3	0.5	500 : 25	3	201±3.24	0.68±0.02	1.2±0.10
4	0.5	500 : 37.5	3	200±2.99	0.81±0.07	1.5±0.00
5	0.5	500 : 50	3	203±1.60	0.69±0.02	3.4±0.12
6	2	500 : 0	3	200±5.85	0.74±0.08	0
7	2	500 : 12.5	3	214±3.82	0.71±0.07	0.8±0.00
8	2	500 : 25	3	215±5.82	0.71±0.04	1.6±0.32
9	2	500 : 37.5	3	219±2.06	0.70±0.09	1.2±0.06
10	2	500 : 50	3	219±1.17	0.69±0.09	3.2±0.00

Data are expressed as the mean ± SD (n=3 each)

References

1. Sugaya, T. *et al.* Angiotensin II type 1a receptor-deficient mice with hypotension and hyperreninemia. *J. Biol. Chem.* **270,** 18719–18722 (1995).
2. Ishibashi, M. *et al.* Bone Marrow-Derived Monocyte Chemoattractant Protein-1 Receptor CCR2 Is Critical in Angiotensin II-Induced Acceleration of Atherosclerosis and Aneurysm Formation in Hypercholesterolemic Mice. *Arterioscler. Thromb. Vasc. Biol.* **24,** e174–e178 (2004).
3. Matsui, Y. *et al.* Distinct Roles of Autophagy in the Heart During Ischemia and Reperfusion: Roles of AMP-Activated Protein Kinase and Beclin 1 in Mediating Autophagy. *Circ. Res.* **100,** 914–922 (2007).
4. Hsu, C. P. *et al.* Silent Information Regulator 1 Protects the Heart From Ischemia/Reperfusion. *Circulation* **122,** 2170–2182 (2010).
5. Kawashima, Y., Yamamoto, H., Takeuchi, H., Hino, T. & Niwa, T. Properties of a peptide containing DL-lactide/glycolide copolymer nanospheres prepared by novel emulsion solvent diffusion methods. *Eur. J. Pharm. Biopharm.* **45,** 41–48 (1998).
6. Katsuki, S. *et al.* Nanoparticle-Mediated Delivery of Pitavastatin Inhibits Atherosclerotic Plaque Destabilization/Rupture in Mice by Regulating the Recruitment of Inflammatory Monocytes. *Circulation* **129,** 896–906 (2014).
7. Murakami, H., Kobayashi, M., Takeuchi, H. & Kawashima, Y. Preparation of poly(DL-lactide-co-glycolide) nanoparticles by modified spontaneous emulsification solvent diffusion method. *Int. J. Pharm.* **187,** 143–152 (1999).

8. Tsukada, Y. *et al.* Particle size control of poly(dl-lactide-co-glycolide) nanospheres for sterile applications. *Int. J. Pharm.* **370,** 196–201 (2009).

9. Swirski, F. K. *et al.* Identification of splenic reservoir monocytes and their deployment to inflammatory sites. *Science* **325,** 612–616 (2009).

10. Takagi, H. *et al.* Activation of PKN mediates survival of cardiac myocytes in the heart during ischemia/reperfusion. *Circ. Res.* **107,** 642–649 (2010).

11. Lal, H. *et al.* Glycogen synthase kinase-3α limits ischemic injury, cardiac rupture, post-myocardial infarction remodeling and death. *Circulation* **125,** 65–75 (2012).

12. Vasquez, K. O., Casavant, C. & Peterson, J. D. Quantitative Whole Body Biodistribution of Fluorescent-Labeled Agents by Non-Invasive Tomographic Imaging. *PLoS ONE* **6,** e20594 (2011).

13. Nagoshi, T. *et al.* PI3K rescues the detrimental effects of chronic Akt activation in the heart during ischemia/reperfusion injury. *J. Clin. Invest.* **115,** 2128–2138 (2005).

14. Fullmer, T. M. *et al.* Insulin suppresses ischemic preconditioning-mediated cardioprotection through Akt-dependent mechanisms. *J. Mol. Cell. Cardiol.* **64,** 20–29 (2013).

15. Howard, M. D., Greineder, C. F., Hood, E. D. & Muzykantov, V. R. Endothelial targeting of liposomes encapsulating SOD/catalase mimetic EUK-134 alleviates acute pulmonary inflammation. *J. Control Release* **177,** 34–41 (2014).

16. Han, K. H. *et al.* HMG-CoA reductase inhibition reduces monocyte CC chemokine receptor 2 expression and monocyte chemoattractant protein-1-mediated monocyte recruitment in vivo. *Circulation* **111,** 1439–1447 (2005).

17. Litt, M. R., Jeremy, R. W., Weisman, H. F., Winkelstein, J. A. & Becker, L. C. Neutrophil depletion limited to reperfusion reduces myocardial infarct size after 90 minutes of ischemia. Evidence for neutrophil-mediated reperfusion injury. *Circulation* **80,** 1816–1827 (1989).

18. Romson, J. L. *et al.* Reduction of the extent of ischemic myocardial injury by neutrophil depletion in the dog. *Circulation* **67,** 1016–1023 (1983).

19. Jolly, S. R. *et al.* Reduction of myocardial infarct size by neutrophil depletion: effect of duration of occlusion. *Am. Heart J.* **112,** 682–690 (1986).

20. Chatelain, P. *et al.* Neutrophil accumulation in experimental myocardial infarcts: relation with extent of injury and effect of reperfusion. *Circulation* **75,** 1083–1090 (1987).

21. Granfeldt, A. *et al.* Neutrophil inhibition contributes to cardioprotection by postconditioning. *Acta Anaesthesiol Scand* **56,** 48–56 (2012).

22. de Lorgeril, M. *et al.* Influence of leukopenia on collateral flow, reperfusion flow, reflow ventricular fibrillation, and infarct size in dogs. *Am. Heart J.* **117,** 523–532 (1989).

23. Arai, M. *et al.* An anti-CD18 antibody limits infarct size and preserves left ventricular function in dogs with ischemia and 48-hour reperfusion. *J. Am. Coll. Cardiol.* **27,** 1278–1285 (1996).

24. Tanaka, M. *et al.* Effect of anti-CD18 antibody on myocardial neutrophil accumulation and infarct size after ischemia and reperfusion in dogs. *Circulation* **87,** 526–535 (1993).

25. Williams, F. M., Kus, M., Tanda, K. & Williams, T. J. Effect of duration of ischaemia on reduction of myocardial infarct size by inhibition of neutrophil accumulation using an anti - CD18 monoclonal antibody. *Br. J. Pharmacol.* **111,** 1123–1128 (1994).

26. Ma, X. L., Tsao, P. S. & Lefer, A. M. Antibody to CD-18 exerts endothelial and cardiac protective effects in myocardial ischemia and reperfusion. *J. Clin. Invest.* **88,** 1237–1243 (1991).

27. Simpson, P. J. & Todd, R. F. Reduction of experimental canine myocardial reperfusion injury by a monoclonal antibody (anti-Mo1, anti-CD11b) that inhibits leukocyte adhesion. *J. Clin. Invest.* **81,** 624 (1988).

28. Simpson, P. J. *et al.* Sustained limitation of myocardial reperfusion injury by a monoclonal antibody that alters leukocyte function. *Circulation* **81,** 226–237 (1990).

29. Hartman, J. C. *et al.* Protection of ischemic/reperfused canine myocardium by CL18/6, a monoclonal antibody to adhesion molecule ICAM-1. *Cardiovasc. Res.* **30,** 47–54 (1995).

30. Zhao, Z. Q. *et al.* Monoclonal antibody to ICAM-1 preserves postischemic blood flow and reduces infarct size after ischemia-reperfusion in rabbit. *J. Leukoc. Biol.* **62,** 292–300 (1997).

31. Vakeva, A. P. *et al.* Myocardial infarction and apoptosis after myocardial ischemia and reperfusion: role of the terminal complement components and inhibition by anti-C5 therapy. *Circulation* **97,** 2259–2267 (1998).

32. Lefer, D. J., Flynn, D. M. & Buda, A. J. Effects of a monoclonal antibody directed against P-selectin after myocardial ischemia and reperfusion. *Am. J. Physiol. Heart Circ. Physiol.* **270,** H88–H98 (1996).

33. Gill, E. A., Kong, Y. & Horwitz, L. D. An oligosaccharide sialyl-Lewisx analogue does not reduce myocardial infarct size after ischemia and reperfusion in dogs. *Circulation* **94,** 542–546 (1996).

34. Kilgore, K. *et al.* Reduction of Myocardial Infarct Size in the Rabbit by a Carbohydrate Analog of Sialyl Lewis(x). *J. Cardiovasc. Pharmacol. Ther.* **1,** 49–56 (1996).

35. Birnbaum, Y., Patterson, M. & Kloner, R. A. The effect of CY1503, a sialyl Lewisx analog blocker of the selectin adhesion molecules, on infarct size and 'no-reflow' in the rabbit model of acute myocardial infarction/reperfusion. *J. Mol. Cell. Cardiol.* **29,** 2013–2025 (1997).

36. Lefer, D. J., Flynn, D. M., Phillips, M. L., Ratcliffe, M. & Buda, A. J. A novel sialyl LewisX analog attenuates neutrophil accumulation and myocardial necrosis after ischemia and reperfusion. *Circulation* **90,** 2390–2401 (1994).

37. Silver, M. J. *et al.* Adjunctive selectin blockade successfully reduces infarct size beyond thrombolysis in the electrolytic canine coronary artery model. *Circulation* **92,** 492–499 (1995).

38. Crawford, M. H. *et al.* Complement and neutrophil activation in the pathogenesis of ischemic myocardial injury. *Circulation* **78,** 1449–1458 (1988).

39. Romson, J. L. *et al.* The effect of ibuprofen on accumulation of indium-111-labeled platelets and leukocytes in experimental myocardial infarction. *Circulation* **66,** 1002–1011 (1982).

40. Allan, G., Bhattacherjee, P., Brook, C. D., Read, N. G. & Parke, A. J. Myeloperoxidase activity as a quantitative marker of polymorphonuclear leukocyte

accumulation into an experimental myocardial infarct--the effect of ibuprofen on infarct size and polymorphonuclear leukocyte accumulation. *J. Cardiovasc. Pharmacol.* **7,** 1154–1160 (1985).

41. Mullane, K. M., Read, N., Salmon, J. A. & Moncada, S. Role of leukocytes in acute myocardial infarction in anesthetized dogs: relationship to myocardial salvage by anti-inflammatory drugs. *J. Pharmacol. Exp. Ther.* **228,** 510–522 (1984).

42. Hayward, R., Campbell, B., Shin, Y. K., Scalia, R. & Lefer, A. M. Recombinant soluble P-selectin glycoprotein ligand-1 protects against myocardial ischemic reperfusion injury in cats. *Cardiovasc. Res.* **41,** 65–76 (1999).

43. Yang, J. *et al.* Endothelial cell overexpression of fas ligand attenuates ischemia-reperfusion injury in the heart. *J. Biol. Chem.* **278,** 15185–15191 (2003).
